# National and regional prevalence of interpersonal violence from others’ alcohol use: a systematic review and modelling study

**DOI:** 10.1016/j.lanepe.2024.100905

**Published:** 2024-04-17

**Authors:** Carolin Kilian, Sinja Klinger, Jakob Manthey, Jürgen Rehm, Taisia Huckle, Charlotte Probst

**Affiliations:** a Institute for Mental Health Policy Research, Centre for Addiction and Mental Health, 33 Ursula Franklin Street, Toronto, Ontario M5S 2S1, Canada; b Institute of Clinical Psychology and Psychotherapy, Technische Universität Dresden, Chemnitzer Straße 46, Dresden 01187, Germany; c Centre for Interdisciplinary Addiction Research, Department of Psychiatry, University Medical Center Hamburg-Eppendorf, Martinistraße 52, Hamburg 20246, Germany; d Department of Psychiatry, Medical Faculty, University of Leipzig, Semmelweisstraße 10, Leipzig 04103, Germany; e Department of Psychiatry, University of Toronto, 250 College Street, Toronto, Ontario M5T 1R8, Canada; f Campbell Family Mental Health Research Institute, Centre for Addiction and Mental Health, 33 Ursula Franklin Street, Toronto, Ontario M5S 2S1, Canada; g Dalla Lana School of Public Health, University of Toronto, 155 College Street, Toronto, Ontario M5T 3M7, Canada; h Faculty of Medicine, Institute of Medical Science, University of Toronto, Medical Sciences Building, 1 King’s College Circle, Toronto, Ontario M5S 1A8, Canada; i Program on Substance Abuse & WHO CC, Public Health Agency of Catalonia, Barcelona, Spain; j Social and Health Outcomes Research and Evaluation & Whariki Research Centre, Massey University, 90 Symonds Street, Auckland 1010, New Zealand; k Heidelberg Institute of Global Health (HIGH), Medical Faculty and University Hospital, Heidelberg University, Im Neuenheimer Feld 130.3, Heidelberg 69120, Germany

**Keywords:** Alcohol consumption, Interpersonal violence, Intimate partner violence, Alcohol’s harm to others, Public health

## Abstract

**Background:**

While alcohol use is an established risk factor for interpersonal violence, the extent to which people are affected by interpersonal violence from others’ drinking has not yet been quantified for different world regions. This modelling study aims to provide the first estimates of the national and regional prevalence of interpersonal violence from others’ drinking.

**Methods:**

An international systematic literature search (02/28/2023, Prospero: CRD42022337364) was conducted to identify general adult population studies assessing the prevalence of interpersonal violence from others’ drinking with no restrictions to publication date or language. Reports that did not provide data on interpersonal violence from others’ drinking (primary outcome), were no original research studies, or captured a selected group of people only, were excluded. Observed prevalence data were extracted and used to build fractional response regression models to predict past-year prevalence of emotional and physical violence from others’ drinking in 2019. Random-effects meta-regression models were used to aggregate the observed prevalence of sexual and intimate partner violence. Study risk of bias (ROB) was assessed using a modified version of the Newcastle–Ottawa Scale.

**Findings:**

Out of 13,835 identified reports, 50 were included covering just under 830,000 individuals (women: 347,112; men: 322,331; men/women combined: 160,057) from 61 countries. With an average prevalence of 16·8% (95% CI: 15·2–18·3%) and 28·3% (95% CI: 23·9–32·4%) in men and women combined in the GBD super regions High Income and Central Europe, Eastern Europe, & Central Asia, respectively, emotional violence was the most common form of interpersonal violence from others’ drinking. Physical violence averaged around 3% (women) and 5% (men) in both regions. The pooled prevalence of sexual violence from others’ drinking in men and women was 1·3% (95% CI: 0·5–3·3%, 95% PI: 0·1–16·9%) and 3·4% (95% CI: 1·4–8·3%, 95% PI: 0·2–35·3%), respectively, and ranged between 0·4% (95% CI: 0·1–1·6%, 95% PI: 0·0–7·3%) and 2·7% (95% CI: 1·1–6·3%, 95% PI: 0·2–30·0%) for different forms of intimate partner violence. ROB was moderate or critical for most reports; accounting for critical ROB did not substantially alter our results.

**Interpretation:**

The share of the population experiencing harms from others’ drinking is significant and should be an integral part of public health strategies.

**Funding:**

Research reported in this publication was supported by the Canadian Institutes of Health Research (CIHR; grant: CIHR FRN 477887).

**Research in context**

**Evidence before this study**

Despite the established link between alcohol use and interpersonal violence, the perspective of those affected by this violence is insufficiently captured. Interpersonal violence from others’ drinking refers to any form of interpersonal violence (e.g., emotional or physical), in which the person committing the violence has consumed alcohol, regardless of whether the person affected by the violence has used alcohol or not. A systematic search in Web of Science, Medline, PsycInfo, Embase, and PubMed (search terms, example for PubMed: ((interpersonal violence [tiab] OR harm from other∗[tiab] OR second?hand harm [tiab]) AND (alcohol [tiab] OR drinking [tiab]) AND (frequency [tiab] OR prevalence [tiab])) AND (systematic review [pt] OR meta?analysis [pt])) revealed that no systematic reviews or meta-analyses have been published by 03/26/2024 providing estimates on the prevalence of interpersonal violence from others’ drinking in the general adult population for Europe or other world regions.

**Added value of this study**

This systematic review and modelling study is the first to provide national and regional estimates of interpersonal violence from others’ drinking for the Global Burden of Disease super regions High Income and Central Europe, Eastern Europe, & Central Asia. We estimated that about every sixth adult in the High Income region, and about every fourth adult in the region of Central Europe, Eastern Europe, & Central Asia experienced past-year emotional violence from another person’s drinking in 2019 (men and women combined). The average regional prevalence of physical violence from others’ drinking averaged in both regions at 3·3% and 5·3–5·4% in women and men, respectively. Sexual violence and intimate partner violence from others' drinking were rarely assessed in the identified literature, preventing us from estimating their regional prevalence.

**Implications of all the available evidence**

On the assumption of alcohol’s causal contribution to interpersonal violence, our modelling study demonstrates that a considerable share of the population in Europe and beyond is affected by interpersonal violence from others' drinking. Alcohol policy and individual- and family-level interventions should address the full range of health harms caused by alcohol, including interpersonal violence from others’ drinking in general and alcohol-involved intimate partner violence in particular. This holds particularly true for European countries, which continue to be those with the highest alcohol consumption levels globally.

## Introduction

Interpersonal violence is a major threat to global health. In 2019, more than 310 million cases of interpersonal violence occurred globally, and 415,000 people lost their lives due to interpersonal violence.^1^ Interpersonal violence, that is, the intentional use of power or physical force against another person or group which is highly likely to adversely affect them,^2^ is among the leading causes of death among 15- to 49-year-old men and hinders progress towards gender equality.^1^^,^^3^ While the aetiology of interpersonal violence is inherently complex, with various individual and societal factors at play, alcohol is an established risk factor^4^, ^5^, ^6^ and recognised as an integral part of violence prevention strategies.^7^

Alcohol has a stronger relationship with aggression than any other psychoactive substance.^8^ The dose–response relationship appears to follow an inverse U-shape, with an accelerated increase in the risk of aggressive behaviour with increasing blood alcohol concentrations (BAC), before dropping off at very high BAC.^6^^,^^9^ Alcohol-induced decreases in the inhibitory control and impairments in decision-making processes and the processing of emotions are considered key neurobiological pathways to aggression.^5^ However, other violence-impelling factors and socio-cultural norms play a pivotal role in exhibiting violence.^4^ Conservative estimates of the alcohol-attributable fraction of violence-related injuries suggest a causal effect of alcohol in 14·9% of the incidents globally (for comparison, see^10^).^11^ In other words, one in six injuries from interpersonal violence could have been avoided if no alcohol were consumed.

Previous reviews on this topic have largely focussed on the risk of being exposed to or committing interpersonal violence associated with alcohol use,^6^ as well as the potential effectiveness of alcohol policy interventions to address such violence.^12^^,^^13^ Despite the established link between alcohol use and committing interpersonal violence,^4^, ^5^, ^6^ no estimates are yet available on the regional or global prevalence of interpersonal violence from others’ drinking. However, it is of critical importance to assess the extent to which individuals are affected by interpersonal violence from others’ drinking in order to determine prevention needs. This systematic review and modelling study therefore aims to estimate the national, regional, and global prevalence of interpersonal violence from others’ drinking for the first time.

For this purpose, we defined interpersonal violence from others’ drinking as reporting at least one event of interpersonal violence within a defined period (e.g., past 12 months), in which the person committing the violence (henceforth: aggressor) has consumed alcohol, regardless of whether the person affected by the act of violence (henceforth: victim) consumed alcohol or not. Our investigation is restricted to non-fatal acts of violence in adulthood, and we distinguish four forms of interpersonal violence, that is, physical (e.g., physical assaults or fights), sexual (e.g., sexual harassment, assault, or rape), and emotional violence (e.g., verbal abuse, humiliation, or threats), as well as any intimate partner violence (Supplement S1). Intimate partner violence is any physical, emotional, or sexual violence experienced from a current or former partner.^2^

## Methods

This systematic review is reported in accordance with the Preferred Reporting Items for Systematic Reviews and Meta-Analyses (PRISMA, Supplement S2).^14^ The review protocol was preregistered at Prospero (CRD42022337364). For a discussion of the actual implementation of the study protocol, see Supplement S3.

### Search strategy and selection criteria

An initial systematic literature search was conducted on June 7, 2022, and updated on February 28, 2023, to identify original research reports providing prevalence data on interpersonal violence from others’ drinking in the general adult population (Supplement S4). We searched the following databases: Web of Science; Medline, PsycInfo, and Embase (Ovid); and PubMed. No restrictions were placed on publication date or language. The systematic search was complemented by a grey literature search (Supplement S5) and a manual screening of the reference lists from published meta-analyses. Once duplicates were identified and excluded by the review platform Covidence,^15^ titles and abstracts, followed by the full-texts were screened by two independent reviewers (CK, SK). Study selection was based on predefined inclusion criteria (Supplement S6), including (1) general population sample of mainly adults (i.e., selective groups of persons such as college students were excluded), (2) assessing interpersonal violence from others’ drinking (for the definition, see above), and (3) reporting prevalence data. We excluded reports that assessed harm other than interpersonal violence as defined in our study (e.g., financial harm), did not link the violence to another person’s drinking, were not original quantitative research studies, or sampled a selected group of people only (e.g., college students). Conflicting decisions were discussed between the reviewers. There was a moderate to substantial agreement in the abstract and full text screening (Cohen’s kappa: 0·60–0·65). Relevant data (i.e., study characteristics, study location, year, sample, outcome, victim-aggressor relationship, prevalence, weighting) from eligible reports were extracted by CK and cross-checked by SK.

### Risk of bias assessment

Risk of bias (ROB) was evaluated using the Newcastle–Ottawa Scale (NOS) for assessing the quality of nonrandomised, cross-sectional studies in meta-analyses,^16^ adapted for the purpose of this review (Supplement S7). The modified NOS included core elements similar to ROB scales that are specific to prevalence studies (e.g., sample representativeness, non-response bias, outcome assessment), while allowing for an assessment on three dimensions (selection, comparability, outcome). Some specific aspects that may be considered relevant for a study’s ROB but were not included in the NOS were entered into the regression models, as outlined below (e.g., sampling, reference period, see Supplement S8). Each study’s ROB was evaluated by two independent reviewers (CK, SK) with substantial agreement (Cohen’s kappa: 0·82). Disagreements were jointly discussed, until a consensus was reached.

### Preparing the data

Several steps were required to prepare the extracted data for the statistical analysis. We first removed data duplicates, i.e., all observations that either based on the same sample or reflect the same outcome in one study. For example, two reports may have employed the same underlying study (i.e., same sample), or provided data on multiple indicators of the same form of interpersonal violence (e.g., for physical violence: being pushed, being hit, physical fight). For each study and outcome, we selected the observation that was based on the highest sample size and reflected the highest prevalence. The latter rule was based on the assumption that someone who has been involved in a physical fight (expected to be less prevalent) has also been hit or pushed in that situation (expected to be more prevalent). This approach also accounts for the fact that interpersonal violence is generally underreported in surveys.^17^ Next, we removed observations referring to women and men combined when sex/gender-specific estimates were available for the same study and outcome. The resulting dataset comprised unique observations from independent samples, with the possibility of multiple observations per country.

Some reports provided the prevalence of interpersonal violence from others’ drinking among alcohol users only. To establish comparability, we estimated the prevalence within the total population, including both alcohol users and non-users, for these reports (*P*_*IV*_, formula 1):

$${\text{Formula}\;1:P}_{IV}=P_{IV\;Drinker}*P_{Alc}+P_{IV\;Drinker}*\frac{1}{{RR}_{Alc}}\left({1-P}_{Alc}\right)$$

The information on the prevalence of interpersonal violence from others’ drinking among current alcohol users (*P*_*IV Drinker*_) and the prevalence of alcohol use in the sample (*P*_*Alc*_) were obtained from the relevant report, while the relative risk of experiencing interpersonal violence after having one drink of alcohol (*RR*_*Alc*_) was taken from the literature (conservative estimate).^9^

### Data analysis

The national prevalence of physical and emotional interpersonal violence from others’ drinking was estimated by fitting fractional response regression models—a special case of logistic regression models suitable for modelling proportional outcomes—to the observed prevalence from relevant reports. For sexual and intimate partner violence, we did not identify sufficient data to apply a similar approach. We therefore combined the observed prevalence of sexual and intimate partner violence from others’ drinking by means of meta-regression. All statistical analyses were conducted in R version 4.2.1 using the packages *stats* (version 3.6.2, generalised linear models) and *metafor* (version 3.8-1, meta-regression).^18^^,^^19^ Figures and maps were produced using *ggplot2* (version 3.4.4)^20^ and *rnaturalearth* (version 1.0.1).^21^

#### Modelling the national and regional prevalence of physical and emotional violence from others’ drinking

For the modelling of both outcomes, we first established a baseline model each using fractional response regression models, including regional identifiers based on the Global Burden of Disease (GBD) study’s region (for regional grouping, see Supplement S9), as well as study variables such as sampling design and ROB (see Supplement S8 ‘study variables’). Study variables were entered stepwise and retained in the baseline models if they contributed substantially to the outcome variance, defined as a relative increase in *R*^*2*^ of ≥5%. Next, potential covariates were explored in the fitted baseline model for each outcome. The selection of potential covariates was based on prior evidence and included indicators of alcohol consumption,^6^ economic wealth,^22^ and demographic factors (see Supplement S8 ‘covariates’).^23^ Covariates with a positively skewed non-normal distribution (i.e., mean larger median; long tail to the right) identified through visual inspection were log-transformed to reduce the disproportional impact of very high values (e.g., proportion of 15-to-24-year-olds within the total population). For indicators of alcohol use, we further tested their interactions with the GBD regions, as we suspected that their impact on interpersonal violence from others’ drinking may vary across regions. Covariates were included in the regression models applying the same criterion as outlined above (relative increase in *R*^*2*^ of ≥5%).

The final fractional response regression models were then built by including all relevant covariates in the fitted baseline models for each outcome using a stepwise approach and employing the same criterion for substantial contribution to variance explanation. Graphical inspection of the outcome residuals over time was carried out to examine secular trends (see Supplement S10). As some data were collected in the years 2020/2021, we further explored a potential impact of the COVID-19 pandemic using a subsample of our data, including studies from those countries where at least two measurements—one before and one in 2020 or later—were available. Using adjusted multi-level fractional response regression models, we found no significant COVID impact on the prevalence of either physical or emotional violence from others’ drinking (for details on methodology and results, see Supplement S11). Thus, no covariate flagging the years of the COVID-19 pandemic was included in the final model. All models were weighted using each study’s inverse variance weight extracted from a random-effects meta-analysis applying a logit transformation for proportions and using the DerSimonian-Laird estimator.

Finally, we used the fitted regression models to predict the country-specific past-year prevalence of physical and emotional from others’ drinking in 2019 by reading in the country- and year-specific covariate data. The year 2019 was chosen as the most recent year with complete covariate data. We only modelled GBD super regions where data from at least 25% of the region’s countries were available. The regional prevalence was computed as the population-weighted average of the estimated country-specific prevalence using population data (15–64) from the United Nations population prospects.^24^ We used bootstrapping drawing 1000 samples to estimate the 95% confidence intervals (CI), considering the uncertainty based on the predicted standard error.

#### Aggregating the prevalence of sexual and intimate partner violence from others’ drinking

Random-effects meta-regression models using restricted maximum-likelihood estimation were used to combine the observed prevalence data for sexual and intimate partner violence separately. Meta-regression models were preferred over simple random-effects meta-analyses to account for the clustered nature of the data^25^ and relevant covariates. The study identifier was included as random intercept. The dependent variable was the logit-transformed observed prevalence of sexual and intimate partner violence from others’ drinking, respectively.

The following covariates were included in the meta-regression models: study sample (men, women, men and women combined), GBD regional identifier, study’s reference period (if applicable), and form of violence (for intimate partner violence only: physical, emotional, sexual). Only those significant were retained in a backward selection process given the small number of observations. To evaluate the pooled prevalence in subsamples, the fitted meta-regression models were recalculated without intercept. Several sensitivity analyses were conducted to evaluate the impact of ROB (critical, non-critical) and studies with limited comparability.

To reflect the between-study heterogeneity in pooled estimates, 95% prediction intervals (PI) were computed using the *predict* function (*metafor* package). We further report Cochran’s *Q* and the *I*^*2*^ statistic for between-study heterogeneity, with *I*^*2*^ > 50% considered substantial heterogeneity. However, as our study combines prevalence data, substantial between-study heterogeneity is to be expected as the prevalence of interpersonal violence from others’ drinking will vary across studies due to true variation in the prevalence across countries, methodological variations (e.g., sampling, interview effects), and residual errors. Potential publication bias was examined based on visual inspections of funnel plots.

### Role of the funding source

Research reported in this publication was supported by the Canadian Institutes of Health Research (CIHR; grant: CIHR FRN 477887). The content is solely the responsibility of the authors and does not necessarily represent the official views of CIHR.

## Results

**Fig. 1:**
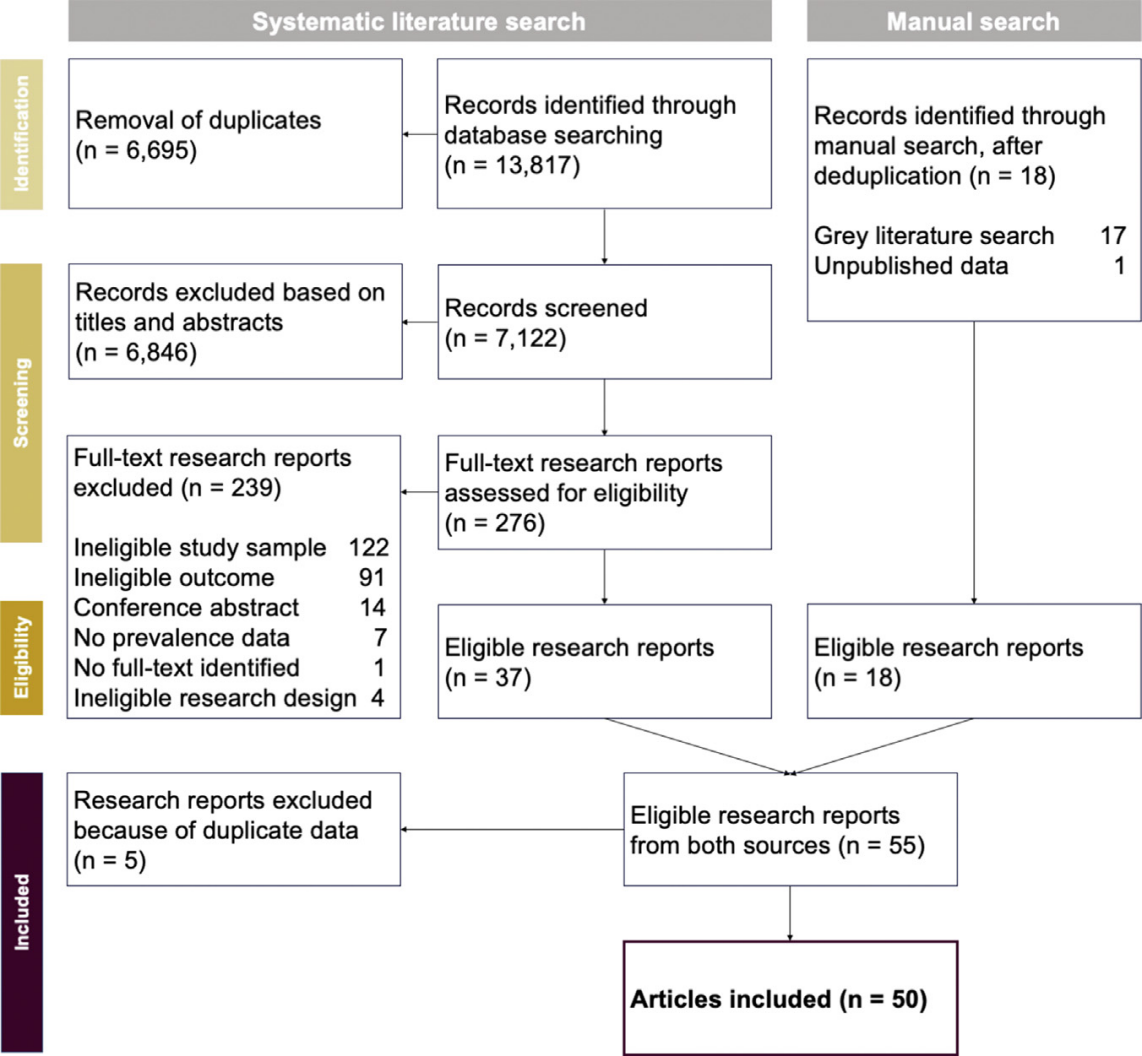
Flowchart of study selection.

Out of 13,817 research reports identified in the systematic literature search, 37 were eligible for inclusion. Another 18 reports were found through the manual search, yielding a total of 55 reports (Fig. 1). After removing those with duplicate data (*n* = 5), 50 reports remained covering 829,500 individuals from more than 60 countries, mostly located in the two GBD regions High Income (*n* = 29) and Central Europe, East Europe, & Central Asia (*n* = 15). Fewer reports were based in Southeast Asia, East Asia, & Oceania (*n* = 9), Latin America & Caribbean (*n* = 5), South Asia (*n* = 2), and Sub-Saharan Africa (*n* = 1). None of the included reports provided data from countries located in North Africa & Middle East. We therefore predicted the prevalence for the two GBD regions High Income and Central Europe, Eastern Europe, & Central Asia only and refrained from estimating the global prevalence. An overview of the included reports and their key characteristics is provided in Table 1.

**Table 1:** Overview of studies included in this systematic literature review and modelling study.

Reference	Country	Year	Study	Sampling	Sample size	Women (%)	Age range	Alcohol’s harm to other: outcome and reference period	Risk of bias (selection/ comparability/ outcome)
Australian Institute of Health and Welfare 2000^a^^,^^c^^,^^26^	Australia	1998	National Drug Strategy Household Survey	Multi-stage stratified sampling	10,030 (past-year alcohol users)	55·4	14+	Physical violence (PY) Emotional violence (PY)	Critical (0/1/1)
Australian Institute of Health and Welfare 2002^a^^,^^27^	Australia	2001	National Drug Strategy Household Survey	Multi-stage stratified sampling	26,744	55·6	14+	Physical violence (PY) Emotional violence (PY)	Moderate (1/1/1)
Australian Institute of Health and Welfare 2005^a^^,^^28^	Australia	2004	National Drug Strategy Household Survey	Multi-stage stratified sampling	29,445	56·4	14+	Physical violence (PY) Emotional violence (PY)	Moderate (1/1/1)
Australian Institute of Health and Welfare 2008^a^^,^^29^	Australia	2007	National Drug Strategy Household Survey	Multi-stage stratified sampling	23,356	56·2	14+	Physical violence (PY) Emotional violence (PY)	Moderate (1/1/1)
Australian Institute of Health and Welfare 2011^a^^,^^30^	Australia	2010	National Drug Strategy Household Survey	Multi-stage stratified sampling	26,648	55·2	14+	Physical violence (PY) Emotional violence (PY)	Moderate (1/2/1)
Australian Institute of Health and Welfare 2014^a^^,^^31^	Australia	2013	National Drug Strategy Household Survey	Multi-stage stratified sampling	23,855	55·5	14+	Physical violence (PY) Emotional violence (PY)	Moderate (1/1/1)
Australian Institute of Health and Welfare 2018^a^^,^^32^	Australia	2016	National Drug Strategy Household Survey	Multi-stage stratified sampling	23,772	54·4	14+	Physical violence (PY) Emotional violence (PY)	Moderate (1/1/1)
Australian Institute of Health and Welfare 2020^a^^,^^33^	Australia	2019	National Drug Strategy Household Survey	Multi-stage stratified sampling	22,274	54·4	14+	Physical violence (PY) Emotional violence (PY)	Moderate (1/2/1)
Basile et al., 2021^34^	USA	2010–2012	National Intimate Partner and Sexual Violence Study	Random sampling (dual-frame random digit dialing (RDD) sample)	41,174	54·9	18–99	Sexual violence (LT)	Moderate (1/2/1)
Beckhoff et al., 2022^a^^,^^35^	Denmark	2011	National Danish Alcohol and Drug Survey	Random sampling	4849	52·5	15–79	Physical violence (PY) Emotional violence (PY)	Critical (1/1/0)
Beynon et al., 2019^a^^,^^36^	United Kingdom^b^	2015–2016	Alcohol Toolkit Survey	Random sampling/Quota sampling (hybrid of random probability sampling and simple quota sampling)	4881	51·1	16–99	Physical violence (PY) Emotional violence (PY) Sexual violence (PY) Any violence (PY)	Moderate (1/1/1)
Bryant and Lightowlers 2021^a^^,^^37^	United Kingdom^b^	2013–2018	Crime Survey for England and Wales	Random sampling	174,178	51·1	.	Physical violence (PY)	Moderate (2/1/1)
Casswell et al., 2011^a^^,^^38^	New Zealand	2008–2009	.	Random sampling	3068 (know fairly heavy drinkers)	59·9	12–80	Physical violence (PY) Emotional violence (PY) Sexual violence (PY)	Moderate (1/1/1)
De Oliveira et al., 2009^c^^,^^39^	Brazil	2005–2006	.	Probabilistic sampling	1631	58·8	18+	Physical violence (PY)	Low (2/2/1)
Gell et al., 2015^a^^,^^40^	United Kingdom^b^	2012–2014	GENAHTO	Quota sampling	2027 (1007–1020)	47·8, 50·0	16+, 18+	Physical violence (PY) Emotional violence (PY) Sexual violence (PY)	Critical (1/0/0)
Graham et al., 2011^a^^,^^c^^,^^41^	Argentina^b^, Australia^b^, Belize, Brazil^b^, Canada^b^, Costa Rica^b^, Czechia, Denmark, Iceland, India^b^, Isle of Man, Japan, Kazakhstan^b^, New Zealand, Nicaragua^b^, Nigeria^b^, Peru^b^, Spain^b^, Sweden, United Kingdom^b^, Uruguay^b^, USA	1995–2007	GENACIS	Multi-stage random sampling	53,791 (623–9,815, past-year alcohol users)	47·5 (7·0–100·0)	18–65 (Hungary: 19–65; Japan: 20–65; USA_1_: 21–65 [women only])	Physical violence (PY)	Critical (0/1/0)
Greenfield et al., 2015^a^^,^^42^	USA	2000–2015	National Alcohol Survey	Random sampling	21,184	55·2	18+	Physical violence (PY)	Critical (1/0/1)
Hanh et al., 2019^a^^,^^43^	Vietnam^b^	2017	.	Four-stage cluster sampling by region	2394 (household sample)	.	.	Physical violence (PY) Emotional violence (PY) Any violence (PY)	Moderate (2/1/1)
Health Canada 2008^a^^,^^44^	Canada	2004	Canadian Addiction Survey	Multi-stage random stratified sampling	13,023	59·1	18+	Physical violence (PY) Emotional violence (PY)	Moderate (1/2/1)
Hingson et al., 2001^a^^,^^c^^,^^45^	USA	1992	National Longitudinal Alcohol Epidemiologic Survey	Multi-stage stratified sampling	42,862	.	18+	Physical violence (PY, LT)	Critical (1/0/1)
Hingson et al., 2009^a^^,^^c^^,^^46^	USA	2006	.	Random sampling	3805 (current or former drinkers)	48·9	18–39	Physical violence (LT)	Critical (0/1/1)
Hope et al., 2018^a^^,^^47^	Ireland	2015	Alcohol harm to others population survey	Probabilistic sampling	2005	51·1	18+	Physical violence (PY) Emotional violence (PY)	Moderate (1/2/1)
Jewkes et al., 2013^c^^,^^48^	Bangladesh^b^, Cambodia, China^b^, Indonesia^b^, Papua New Guinea^b^, Sri Lanka^b^	2011–2012	.	Multi-stage random sampling	9951	0·0	18–49	Sexual violence (alcohol-involvement in last rape)	Moderate (1/1/1)
Kaplan et al., 2017^a^^,^^49^	USA	2010	National Alcohol Survey	Random sampling	5885	51·0	18+	Physical violence (PY)	Moderate (1/1/1)
Karriker-Jaffe et al., 2017^a^^,^^50^	USA	2014–2015	National Alcohol Survey	Multi-stage stratified random sampling	5922	52·5	18+	Physical violence (PY) Emotional violence (PY)	Moderate (1/2/1)
Kellner et al., 1996^a^^,^^51^	Canada^b^	1990	Yukon Alcohol and Drug Survey	.	1348	47·2	15+	Physical violence (PY) Emotional violence (PY)	Critical (0/0/0)
Kerr et al., 2021^a^^,^^52^	USA^b^	2016	.	Random sampling	2001	56·5	18+	Physical violence (PY) Emotional violence (PY)	Critical (0/0/1)
Kilian et al., 2020^a^^,^^53^	Austria, Bulgaria, Croatia, Denmark, Estonia, Finland, France, Hungary, Iceland, Lithuania, Norway, Poland, Portugal, Romania, Spain, United Kingdom	2015–2016	RARHA SEAS	Randomized sample selection (mostly multistage stratified probability and simple random sampling)	27,742 (813–3375)	52·4 (50·7–62·3)	18–64	Physical violence (PY) Emotional violence (PY)	Moderate (1/2/1)
Kilian et al., 2023^a^^,^^54^	Austria, Belgium, Bosnia and Herzegovina, Bulgaria, Croatia, Cyprus, Czechia, Denmark, Estonia, Finland, France, Germany, Greece, Hungary, Iceland, Ireland, Italy, Latvia, Lithuania, Luxembourg, Malta, Moldova, Netherlands, Norway, Poland, Portugal, Romania, Serbia, Slovakia, Slovenia, Spain, Sweden, United Kingdom	2021	DEEP SEAS	Non-probabilistic quota sampling	53,798 (1466–3052)	51·0 (49·0–54·0)	18–64	Physical violence (PY) Emotional violence (PY)	Moderate (1/2/1)
Kinjo et al., 2022^a^^,^^55^	Japan	2018	.	Multi-stage random sampling	4627	54·2	20+	Emotional violence (PY) Sexual violence (PY)	Moderate (1/2/1)
Laslett et al., 2017^56^	Australia	2008	Australian Harm to Others Survey	Random sampling	2649	.	18+	Physical violence (PY) Emotional violence (PY) Sexual violence (PY)	Moderate (1/2/1)
Laslett et al., 2019^57^	Australia, New Zealand, Nigeria^b^, Chile^b^, Laos, Thailand, Vietnam, India^b^, Sri Lanka	2008–2014	Australian Harm to Others Survey, WHO/ThaiHealth study	Random sampling	3610 (1394–2216)	50·9 (49·2–54·2)	18+	Emotional violence (PY) Any violence (PY)	Moderate (1/2/1)
Marmet & Gmel 2017^a^^,^^58^	Switzerland	2011–2012	Addiction Monitoring in Switzerland	Multi-stage stratified sampling	2469	56·5	15+	Physical violence (PY) Emotional violence (PY)	Moderate (1/2/1)
Ministry of Health 2007^a^^,^^59^	New Zealand	2003–2004	New Zealand Health Behaviours Survey – Alcohol Use	Random stratified sampling	9847	50·0	12–65	Physical violence (PY) Sexual violence (PY)	Moderate (1/1/1)
Moan et al., 2015^a^^,^^60^	Denmark, Finland, Iceland, Norway, Sweden, United Kingdom^b^	2008–2013	Danish national alcohol and drug survey; Finnish drinking habits survey; Icelandic alcohol survey; Norwegian survey on tobacco and substance use; Habits and consequences – A national survey on tobacco, alcohol and drugs; GENAHTO	Random sampling, quota sampling	22,609 (1249–12,678)	50·0 (48·7–51·3)	18–69	Physical violence (PY) Emotional violence (PY)	Critical (1/0/1)
Moreira et al., 2011^a^^,^^61^	Brazil^b^	2005	.	Multi-stage stratified random sampling	454	44·0	12–65	Physical violence (LT) Emotional violence (LT)	Critical (1/0/0)
Nayak et al., 2019^a^^,^^62^	USA	2014–2015	2015 National Alcohol's Harm to Others Survey; 2015 National Alcohol Survey	Multi-stage stratified sampling	8750	59·3	18+	Physical violence (PY) Emotional violence (PY)	Moderate (1/2/1)
Quigg et al., 2019^a^^,^^63^	United Kingdom^b^	2015	.	Random probability sampling	891	61·5	18+	Physical violence (PY) Emotional violence (PY) Sexual violence (PY)	Critical (1/2/0)
Rossow et al., 1996^a^^,^^c^^,^^64^	Norway	1994	National Opinion Poll	Multi-stage stratified sampling	2711 (alcohol users)	51·0	15+	Physical violence (PY)	Critical (0/0/1)
Rossow & Hauge 2004^a^^,^^65^	Norway	1999	National Opinion Poll	Multi-stage stratified sampling	2170	51·0	15+	Physical violence (PY) Emotional violence (PY)	Moderate (1/1/1)
Scott et al., 1999^a^^,^^66^	USA	1990	National Alcohol Survey	Multi-stage stratified sampling	2058	58·0	18+	Physical violence (LT)	Critical (2/0/0)
Storvoll et al., 2016^a^^,^^67^	Norway	2012	National Survey on Alcohol, Tobacco and Drug use	Random sampling	1947	49·0	16–79	Physical violence (PY) Emotional violence (PY) Sexual violence (PY)	Moderate (1/2/1)
Tamutienė et al., 2017^a^^,^^68^	Lithuania	2014	National Opinion Poll	Multi-stage stratified random sampling	1000	44·9	18+	Physical violence (PY) Emotional violence (PY)	Low (2/2/1)
Tamutienė et al., 2022^a^^,^^69^	Lithuania	2020	RARHA SEAS	Multi-stage stratified probability sampling	1015	50·6	18–64	Physical violence (PY) Emotional violence (PY)	Moderate (1/1/1)
Vichitkunakorn et al., 2019^70^	Thailand	2017	National Cigarette and Alcohol Consumption Survey	Multi-stage stratified random sampling	39,630 (households)	.	15+	Any violence (PY)	Critical (1/0/0)
Waleewong et al., 2017^a^^,^^71^	Thailand	2012–2013	.	Multi-stage stratified random sampling	1695	59·1	18–70	Physical violence (PY) Emotional violence (PY) Sexual violence (PY)	Critical (2/1/0)
Waleewong et al., 2018^a^^,^^72^	Thailand, Sri Lanka, India^b^, Vietnam, Laos	2012–2014	WHO/ThaiHealth study	Multi-stage stratified random sampling	8229 (1212–3284)	50·6 (49·2–52·1)	18–65	Physical violence (PY) Emotional violence (PY)	Moderate (1/1/1)
Wells et al., 2000^a^^,^^73^	Canada^b^	1997	Ontario drug monitor	Stratified sampling	1001	51·9	18+	Physical violence (PY) Emotional violence (PY)	Moderate (1/1/1)
Wichaidit et al., 2020^74^	Thailand	2017	Thailand Smoking and Drinking Behaviour Survey	Multi-stage stratified random sampling	45,296 (households with alcohol users)	.	18+	Physical violence (PY) Emotional violence (PY)	Critical (0/0/1)
Yu et al., 2022^a^^,^^75^	Hong Kong SAR, China	2019	.	Random sampling	3200	53·8	18–74	Physical violence (PY) Emotional violence (PY) Sexual violence (PY)	Critical (1/2/0)

Dots indicated missing information. LT: Lifetime prevalence. PY: Past-year prevalence. USA: United States of America. DEEP SEAS: Developing and Extending Evidence and Practice from the Standard European Alcohol Survey. GENACIS: Gender, Alcohol and Culture: An International Study. GENAHTO: Gender and Alcohol’s Harm To Others. RARHA SEAS: Reducing Alcohol Related Harm – Standard European Alcohol Survey.

a Studies included in modelling interpersonal violence from others’ drinking.

b Data was collected in metropolitan areas or in specific regions or states only.

c Survey participants were interviewed as the aggressors (i.e., whether they had harmed another person in a situation where they had consumed alcohol).

### Risk of bias

The majority of studies had a moderate ROB (*n* = 30), with 18 studies having a critical and 2 studies a low ROB (Table 1). A critical ROB resulted mostly from not accounting for sex/gender and the victim-aggressor relationship (selection domain, *n* = 11), presenting non-weighted prevalence data (comparability domain, *n* = 11), and/or a lack of population representativeness (e.g., sampling restricted to metropolitan areas or alcohol users only) in combination with low response rates (outcome domain, *n* = 7).

### National and regional prevalence of physical violence from others’ drinking

**Fig. 2:**
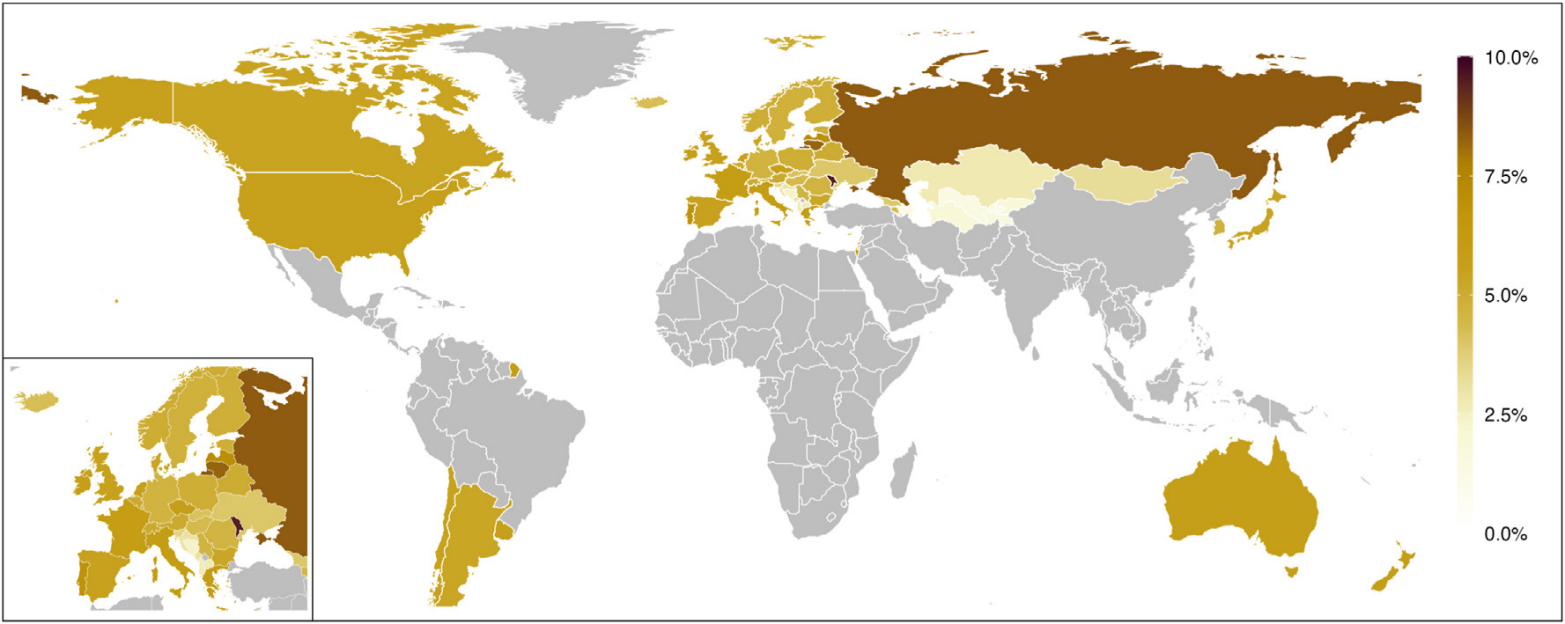
Predicted prevalence of physical violence from others’ drinking reported by men aged 15–64 in the past 12 months in 2019.

The final prediction model for physical violence is depicted in Supplement S12 and explained 55·9% of the outcome variance. Comparing the predicted with the observed prevalence reveal a tendency of our modelling to overestimate very low (≤3%) while underestimating higher prevalences (≥8%; Supplement S13). Table 2, Figs. 2 (men) and 3 (women) show the predicted prevalence of physical violence from others’ drinking for the year 2019. On average, 5·3% (95% CI: 4·3–6·3%) and 3·3% (95% CI: 2·7–4·0%) of men and women in the High Income region and 5·4% (95% CI: 5·0–5·8%) and 3·3% (95% CI: 3·0–3·6%) of men and women in the region of Central Europe, Eastern Europe, & Central Asia were estimated to have experienced past-year physical violence from others’ drinking. In the High Income region, the predicted prevalence was lowest in Malta (men: 3·6%, 95% CI: 2·1–5·2%, women: 2·2%, 95% CI: 1·2–3·2%) and highest in Portugal (men: 6·8%, 95% CI: 4·8–8·8%, women: 4·2%, 95% CI: 2·9–5·5%). For the region of Central Europe, Eastern Europe, & Central Asia, the lowest predicted prevalence was observed in Azerbaijan (men: 1·1%, 95% CI: 0·0–2·5%, women: 0·7%, 95% CI: 0·0–1·5%) and the highest in Moldova (men: 9·5%, 95% CI: 6·7–12·2%, women: 5·9%, 95% CI: 4·0–7·7%).

**Table 2:** Predicted past-year prevalence of having experienced physical and emotional violence from others’ drinking in the general adult population in 2019.

Country	Predicted prevalence of physical violence from others’ drinking (%)		Predicted prevalence of emotional violence from others’ drinking (%, men and women combined)
	Men	Women	
**Central Europe, Eastern Europe, & Central Asia**			
Albania	2·7 (1·2–4·1)	1·6 (0·7–2·5)	23·8 (16·8–30·7)
Armenia	4·5 (0·0–9·3)	2·7 (0·0–5·7)	34·4 (13·7–55·2)
Azerbaijan	1·1 (0·0–2·5)	0·7 (0·0–1·5)	17·8 (6·2–29·3)
Bulgaria	5·0 (2·6–7·4)	3·1 (1·5–4·6)	26·8 (16·7–37·0)
Bosnia and Herzegovina	2·4 (1·0–3·7)	1·4 (0·6–2·3)	24·6 (16·0–33·1)
Belarus	5·3 (3·9–6·6)	3·2 (2·3–4·1)	27·2 (18·8–35·5)
Czechia	3·0 (1·8–4·3)	1·8 (1·1–2·6)	22·1 (16·7–27·5)
Estonia	5·6 (4·1–7·2)	3·4 (2·4–4·5)	23·1 (16·3–29·9)
Georgia	4·9 (3·8–6·0)	3·0 (2·2–3·7)	25·1 (20·3–30·0)
Croatia	3·9 (1·6–6·2)	2·4 (0·9–3·8)	30·2 (20·1–40·3)
Hungary	4·4 (3·2–5·5)	2·7 (1·9–3·4)	24·1 (18·8–29·4)
Kazakhstan	2·8 (0·5–5·0)	1·7 (0·3–3·0)	23·7 (12·6–34·8)
Kyrgyz Republic	1·8 (0·1–3·5)	1·1 (0·1–2·1)	20·7 (10·0–31·4)
Lithuania	7·1 (5·5–8·6)	4·3 (3·3–5·4)	30·9 (25·2–36·5)
Latvia	8·2 (6·1–10·4)	5·1 (3·7–6·5)	31·5 (25·9–37·1)
Moldova	9·5 (6·7–12·2)	5·9 (4·0–7·7)	29·9 (21·0–38·7)
North Macedonia	3·2 (1·7–4·8)	2·0 (1·0–2·9)	25·5 (18·8–32·1)
Montenegro	3·1 (1·6–4·6)	1·9 (1·0–2·8)	27·7 (19·0–36·5)
Mongolia	2·7 (1·4–3·9)	1·6 (0·8–2·4)	25·1 (15·8–34·4)
Poland	4·9 (3·8–6·1)	3·0 (2·2–3·8)	25·1 (19·9–30·3)
Romania	4·6 (3·5–5·7)	2·8 (2·1–3·5)	25·6 (19·9–31·4)
Russian Federation	8·4 (5·9–10·9)	5·2 (3·5–6·9)	33·7 (24·9–42·6)
Serbia	4·6 (3·3–5·9)	2·8 (2·0–3·6)	28·4 (22·7–34·1)
Slovakia	4·1 (3·0–5·2)	2·5 (1·8–3·2)	22·5 (15·4–29·6)
Slovenia	3·5 (2·2–4·8)	2·1 (1·3–3·0)	19·8 (13·0–26·6)
Tajikistan	1·6 (0·2–3·0)	1·0 (0·1–1·8)	20·4 (10·7–30·1)
Turkmenistan	1·9 (0·3–3·5)	1·1 (0·2–2·1)	22·4 (10·1–34·7)
Ukraine	4 (1·9–6·1)	2·4 (1·1–3·7)	26·4 (17·2–35·7)
Uzbekistan	1·3 (0·0–2·6)	0·8 (0·0–1·6)	28·2 (0·0–62·1)
Regional population-weighted average	5·3 (4·3–6·3)	3·3 (2·7–4·0)	28·3 (23·9–32·4)
**High income**			
Argentina	5·3 (4·2–6·4)	3·2 (2·5–4·0)	16·1 (10·7–21·4)
Australia	5·7 (4·8–6·7)	3·5 (2·8–4·2)	18·4 (15·8–20·9)
Austria	5·1 (4·1–6·1)	3·1 (2·4–3·8)	19·3 (16·6–21·9)
Belgium	5·1 (4·1–6·0)	3·1 (2·4–3·7)	19·9 (16·6–23·3)
Brunei Darussalam	5·1 (1·5–8·7)	3·1 (0·8–5·4)	.
Canada	5·4 (4·5–6·3)	3·3 (2·7–4·0)	16·7 (14·3–19·2)
Chile	5·5 (4·4–6·5)	3·3 (2·6–4·1)	15·4 (9·9–21·0)
Cyprus	4·9 (4·1–5·8)	3·0 (2·4–3·6)	18·5 (16·0–20·9)
Denmark	5·3 (4·4–6·2)	3·2 (2·6–3·9)	17·9 (14·9–20·9)
Finland	4·9 (4·0–5·7)	3·0 (2·4–3·6)	18·4 (15·4–21·4)
France	5·9 (4·7–7·0)	3·6 (2·8–4·4)	21·8 (18·7–25·0)
Germany	4·7 (3·5–5·8)	2·8 (2·0–3·6)	18·3 (15·0–21·5)
Greece	5·9 (4·9–6·9)	3·6 (2·9–4·3)	22·4 (15·2–29·7)
Iceland	4·2 (3·0–5·5)	2·6 (1·8–3·4)	15·4 (11·2–19·7)
Ireland	5·7 (3·8–7·7)	3·5 (2·2–4·8)	21·6 (18·3–25·0)
Israel	6·3 (3·3–9·2)	3·8 (2·0–5·7)	12·6 (7·6–17·7)
Italy	5·9 (4·4–7·5)	3·6 (2·6–4·7)	17·0 (13·4–20·7)
Japan	5·4 (4·1–6·7)	3·3 (2·4–4·2)	14·3 (10·8–17·8)
Korea, Rep.	4·7 (3·7–5·8)	2·9 (2·2–3·6)	15·0 (12·3–17·8)
Luxembourg	4·7 (2·5–6·8)	2·8 (1·5–4·2)	17·6 (14·1–21·0)
Malta	3·6 (2·1–5·2)	2·2 (1·2–3·2)	12·2 (8·1–16·4)
Netherlands	5·6 (4·4–6·8)	3·4 (2·6–4·2)	16·8 (13·6–20·1)
New Zealand	5·8 (4·9–6·7)	3·6 (2·9–4·2)	19·2 (16·4–22·0)
Norway	5·0 (3·6–6·3)	3·0 (2·1–3·9)	15·1 (11·8–18·4)
Portugal	6·8 (4·8–8·8)	4·2 (2·9–5·5)	24·0 (19·0–29·0)
Singapore	4·3 (1·2–7·4)	2·6 (0·7–4·5)	.
Spain	5·5 (4·–6·3)	3·3 (2·8–3·9)	19·9 (14·7–25·2)
Sweden	4·8 (3·8–5·7)	2·9 (2·2–3·6)	16·6 (13·9–19·3)
Switzerland	5·1 (4·0–6·2)	3·1 (2·3–3·8)	17·3 (14·9–19·7)
United Kingdom	5·5 (4·7–6·3)	3·4 (2·7–4·0)	18·1 (15·3–21·0)
Uruguay	5·5 (4·5–6·6)	3·4 (2·7–4·1)	18·7 (14·5–23·0)
USA	5·5 (4·4–6·7)	3·4 (2·6–4·2)	15·5 (11·0–19·9)
Regional population-weighted average	5·4 (5·0–5·8)	3·3 (3·0–3·6)	16·8 (15·2–18·3)

95% confidence intervals in brackets. USA: United States of America. Prevalence of emotional violence from others’ drinking was not predicted for Andorra, Brunei Darussalam, and Singapore as data on income inequality (Gini index as of 2019) was missing.

**Fig. 3:**
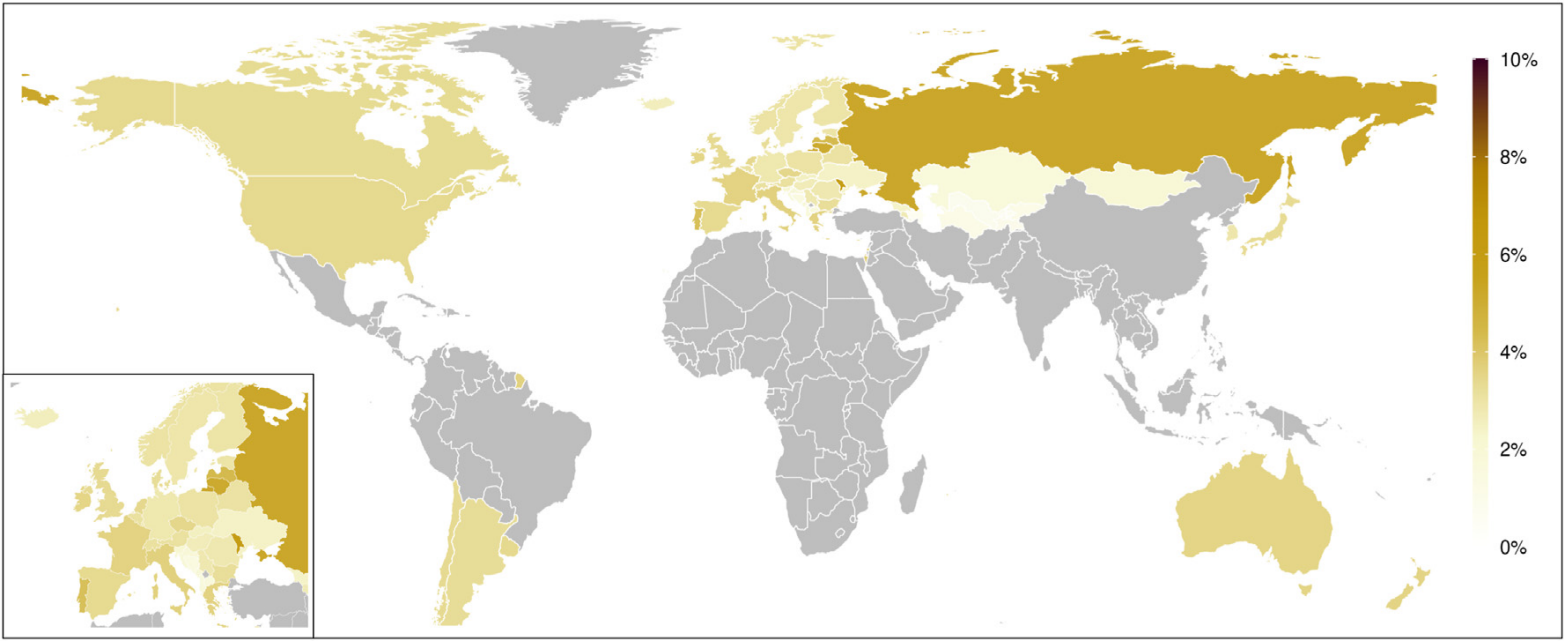
Predicted prevalence of physical violence from others’ drinking reported by women aged 15–64 in the past 12 months in 2019.

The observed past-year prevalence estimates from countries of regions not modelled are available in Supplement S14. In Latin America & Caribbean (Belize, Brazil, Costa Rica, Nicaragua, Peru; 2003–2006), the observed prevalence of physical violence from others’ drinking reported by men and women ranged from 6·0 to 20·7% and 0·8 to 3·8%, respectively. In South Asia (India; 2003–2014), 9·3 to 36·3% of men and 32·7% of women indicated having experienced such violence, while in the countries of Southeast Asia, East Asia & Oceania (Hong Kong, Laos, Sri Lanka, Thailand, Vietnam; 2012–2019), 0·9 to 24·4% of men and 0·2 to 22·4% of women did so. In Nigeria (2003)—the only Sub-Saharan African country with data—the observed prevalence was 2·0% and 2·2% in men and women, respectively.

### National and regional prevalence of emotional violence from others’ drinking

The final prediction model for emotional violence is depicted in Supplement S12 and explained 45·7% of the outcome variance. As the inclusion of the study sample variable (sex/gender) did not substantially contribute to variance explanation (relative change: 3·7%), we did not predict sex-specific prevalence. The comparison of the predicted with the observed prevalence data suggest a tendency towards overestimating prevalence ≤10%, while underestimating prevalence ≥25% (Supplement S15).

**Fig. 4:**
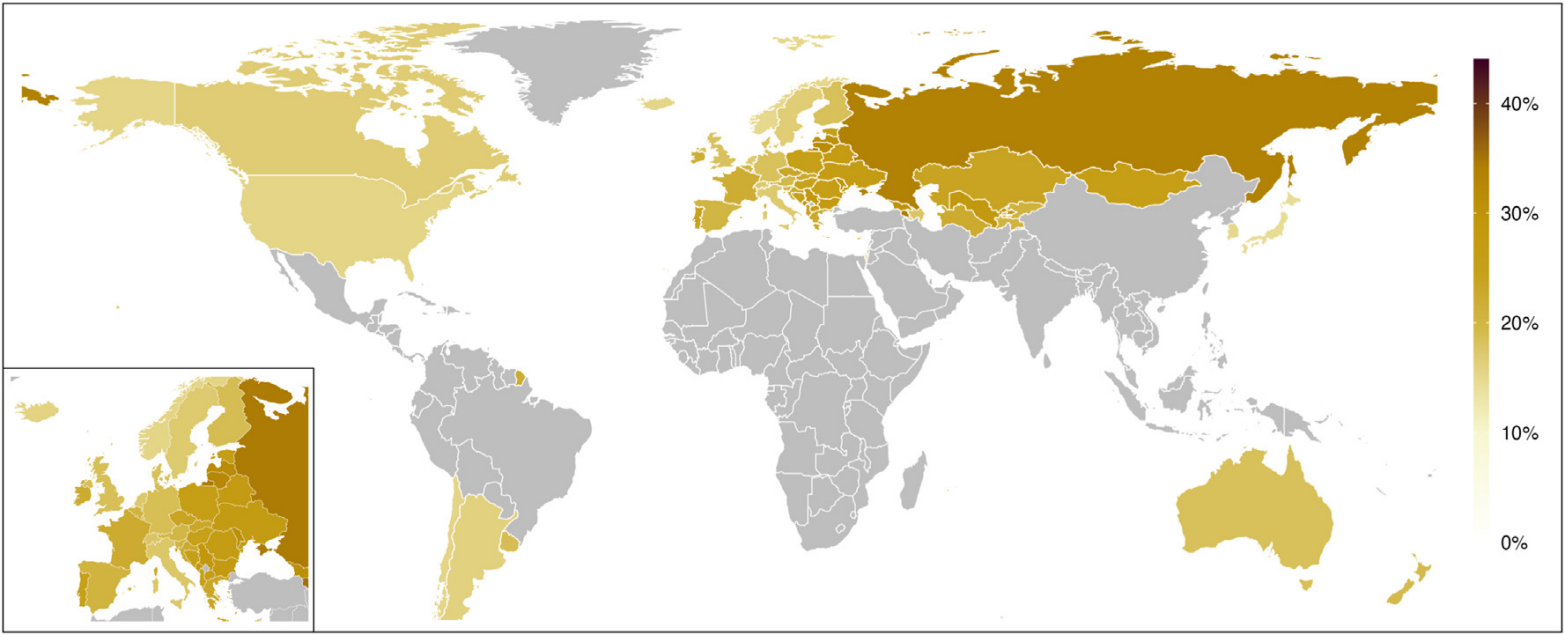
Predicted prevalence of emotional violence from others’ drinking reported by men and women aged 15–64 in the past 12 months in 2019.

The predicted prevalence of past-year emotional violence from others’ drinking in 2019 is shown in Table 2 and Fig. 4. The population-weighted average prevalence was 16·8% (95% CI: 15·2–18·3%) in the High Income region. The lowest prevalence in the High Income region was predicted for Malta (12·2%, 95% CI: 8·1–16·4%) and the highest for Portugal (24·0%, 95% CI: 19·0–29·0%). For countries of the region of Central Europe, Eastern Europe, & Central Asia, the predicted prevalence of emotional violence from others’ drinking ranged between 17·8% (95% CI: 6·2–29·3%) in Azerbaijan and 34·4% (95% CI: 13·7–55·2%) in Armenia.

The observed prevalence from countries of regions that were not modelled is available in Supplement S16. The observed lifetime prevalence of emotional violence from others’ drinking was 7·0% in Latin America & Caribbean (Brazil; 2005) and the past-year prevalence in men and women in South Asia (India; 2014) was 41·5% and 38·0%, respectively. In the countries of Southeast Asia, East Asia & Oceania (Hong Kong, Laos, Sri Lanka, Thailand, Vietnam; 2012–2019), the observed past-year prevalence was 3·7–38·5% among men and 1·7–38·7% among women.

### Sexual violence from others’ drinking

**Fig. 5:**
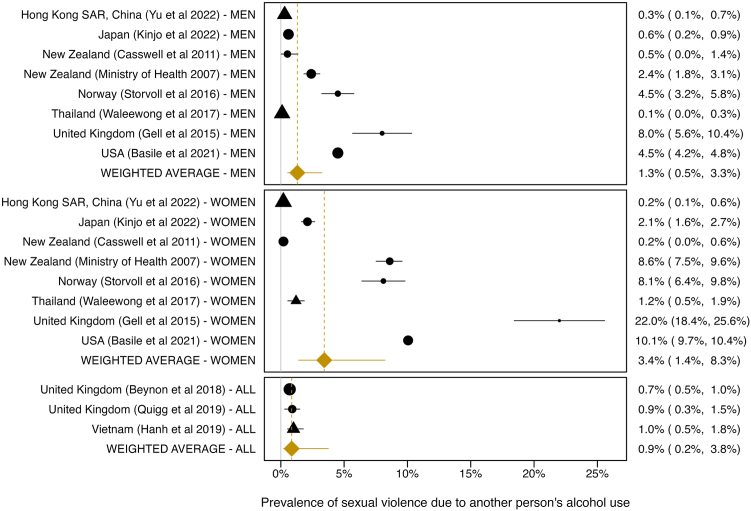
Forest plot depicting the prevalence of sexual violence caused from others' drinking by women, men, and women and men combined (“all”). Circle: High Income; triangle: Southeast Asia, East Asia, & Oceanial; diamond (orange): pooled average. Between-study heterogeneity: *I^^2^^* = 97·6, *Q* = 508·45, *p* < 0·001. 95% prediction intervals accounting for between-study heterogeneity: men: 0·1-16·9%, women: 0·2–35·3%, men and women combined: 0·0–14·6%.

Eleven studies assessed sexual violence from others’ drinking. The pooled prevalence of sexual violence from others’ drinking was 1·3% (95% CI: 0·5–3·3%, 95% PI: 0·1–16·9%) in men and 3·4% (95% CI: 1·4–8·3%, 95% PI: 0·2–35·3%) in women (Fig. 5). Among studies reporting the prevalence for men and women combined, 0·9% (95% CI: 0·2–3·8%, 95% PI: 0·0–14·6%) indicated sexual violence from another person’s drinking. The pooled prevalence was significantly higher in women than in men (*p* = 0·001) and did not differ between studies with and without critical ROB (*p* = 0·621; Supplement S17).

There was substantial between-study heterogeneity and no indication of small-study bias (Supplement S17). Excluding one study with a very high estimate^40^ did marginally reduce the pooled prevalence (men: 1·0%, 95% CI: 0·4–2·5%, women: 2·4%, 95% CI: 1·0–6·1%) with little impact on between-study heterogeneity.

### Intimate partner violence from the partner's drinking

**Fig. 6:**
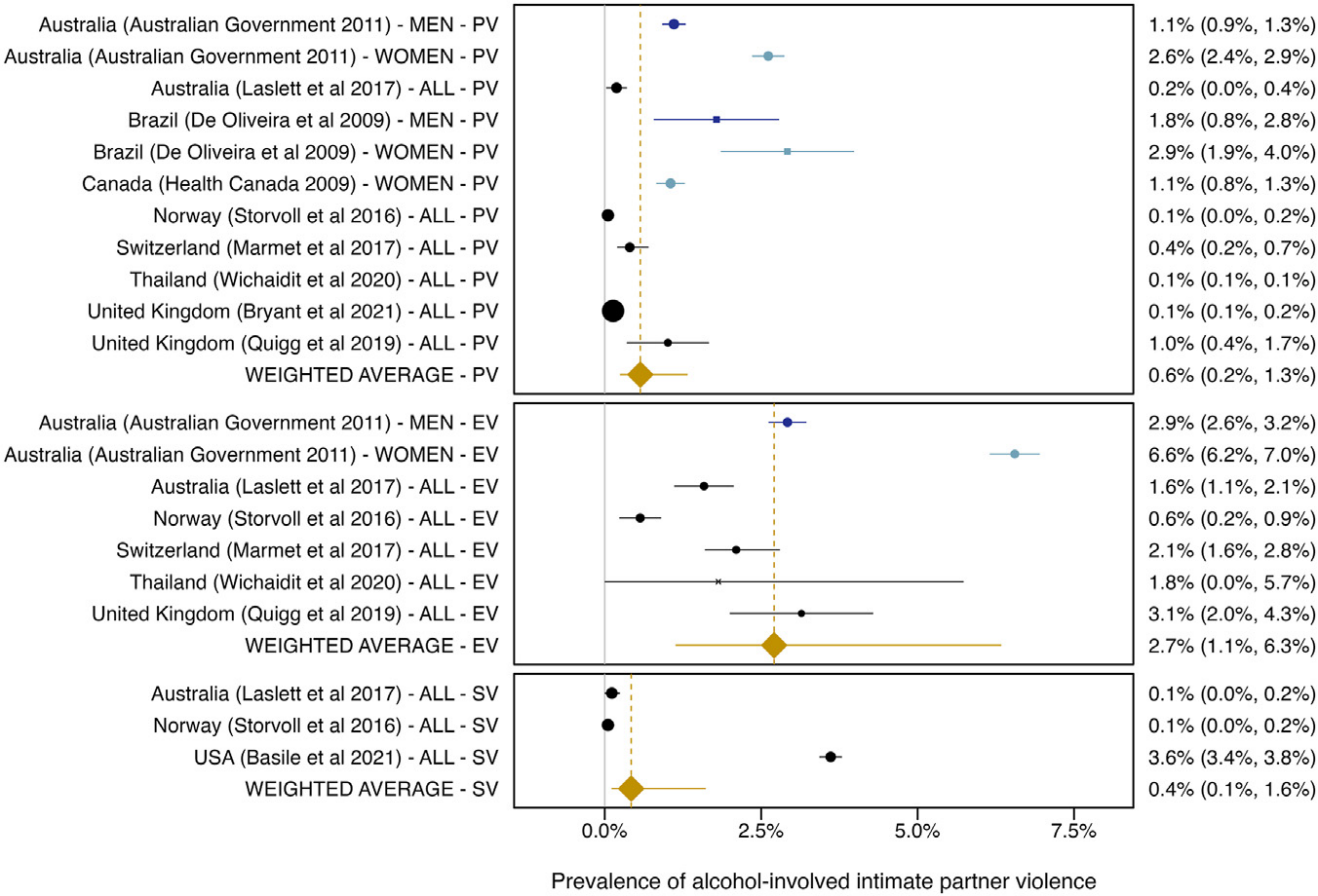
Forest plot depicting the prevalence of intimate partner violence from the partner's alcohol use reported by women (light blue), men (blue), and women and men combined (“all”, black). Circle: High Income; rectangle: Latin America & Caribbean; cross: Southeast Asia, East Asia, & Oceania; diamond (orange): pooled average. Physical violence: PV; emotional violence: EV; sexual violence: SV. Between-study heterogeneity: *I^2^* = 84·4, *Q* = 2207·19, *p* < 0·001. Prediction intervals accounting for between-study heterogeneity: emotional violence: 0·2–30·0%, physical violence: 0·0–8·0%, sexual violence: 0·0–7·3%.

Ten studies reported the prevalence of alcohol-involved intimate partner violence. The pooled prevalence of intimate partner violence from the partner’s drinking was 2·7% (95% CI: 1·1–6·3%, 95% PI: 0·2–30·0%) for emotional violence, 0·6% (95% CI: 0·2–1·3%, 95% PI: 0·0–8·0%) for physical violence, and 0·4% (95% CI: 0·1–1·6%, 95% PI: 0·0–7·3%) for sexual violence (Fig. 6). It should be noted that these estimates reflect the prevalence in the general population, including singles. The prevalence for emotional intimate partner violence was significantly higher than for physical or sexual intimate partner violence (*p* < 0·001 and *p* = 0·004, respectively) and did not differ between studies with and without critical ROB (*p* = 0·730). Excluding studies assessing lifetime prevalence^34^ or interviewing perpetrators^39^ did negligibly alter the pooled estimates. There was substantial between-study heterogeneity and no indication of a small-study bias (Supplement S18).

## Discussion

This systematic review and modelling study is the first to estimate the national and regional prevalence of interpersonal violence from others’ drinking. We estimated that about every fourth adult in the GBD region of Central Europe, Eastern Europe, & Central Asia, and about every sixth in the High Income region experienced past-year emotional violence from another person’s drinking in 2019. The predicted prevalence of having experienced physical violence from others’ drinking was lower, with an average of about 5% in men and 3% in women. Meta-analytic summaries of sexual violence and intimate partner violence from others’ drinking reveal that both forms of interpersonal violence were less common, although their prevalence varied greatly across studies. Our findings clearly demonstrate that alcohol’s health and social burden extends beyond the alcohol user.

Among a variety of other individual and social factors, sex/gender plays a key role in interpersonal violence. In our study, men were found to have a higher prevalence of physical violence from others’ drinking compared to women. As men engage in more physical altercations than women, they may have a higher susceptibility to interpersonal violence including that related to another person’s drinking. Additionally, men are more commonly involved in late-night violence, coinciding with times of elevated alcohol consumption, compared to women.^76^^,^^77^ Regarding emotional violence from others’ drinking, no significant sex/gender difference was observed in our data. Various other studies, including meta-analyses, suggest that men and women exhibit comparable levels of emotional violence, and there is a significant degree of mutual exchange of such violence between them.^78^ Lastly, we found that women experience a higher prevalence of sexual violence associated with someone else’s drinking compared to men, which aligns well with existing evidence.^79^

While researchers in the 1990s already concluded that alcohol may act as a causal contributor to interpersonal violence, this applies only for some individuals under some situations and/or socio-cultural contexts.^4^ Alcohol can contribute to violence by, for example, altering brain receptors and neurotransmission increasing risk taking, impairing frontal brain functions leading to impulsive aggression in vulnerable individuals, alcohol myopia, and impairing problem-solving during conflict.^4^^,^^5^ Interpersonal violence may also increase, however, because alcohol outlets attract substantial crowds of young men who are at risk of violence,^80^ or situational effects, power concerns, or expectations that aggression will be tolerated.^81^ The question therefore remains of how many of the self-reported incidents involving alcohol use were actually caused by alcohol. Or, in other words, using the current common thinking in epidemiology about causality, what proportion of interpersonal violence would disappear in absence of alcohol use.^82^ To our knowledge, there are no systematic studies on how valid such self-reports on causality are. Assuming that these prevalence estimates based on self-reports reflect the actual size of the problem, preventive means should concentrate on the best ways of reducing alcohol consumption, i.e., the WHO “best buys” and best practices outlined in the most recent edition of *Alcohol: No Ordinary Commodity*.^79^

Despite a comprehensive literature search, we were unable to source estimates for large parts of the global population. This observation could be related to both our search strategy, which was limited in the number of databases to be searched and the use of English search terms, and the fact that there are indeed no or insufficient empirical data for countries relatively close to the equator, i.e., countries in Latin America and the Caribbean, countries on the African continent, Middle East, and South(-East) Asia, including the two most populous countries India and China. Given increasing alcohol consumption in some of these regions, we suggest that future studies should address this topic. Our models are also only as good as the input data. We may assume a considerable underreporting of sensitive topics such as violence and this bias may depend on cultural aspects. There are also cross-cultural differences in the willingness to make attributions of alcohol’s involvement in harm, and differences in thresholds of perceived harm.^83^ We were unable to capture such differences, resulting in uncertainty that is not covered in the presented CIs. Moreover, our prediction models tend to overestimate very low and underestimate very high prevalence (model fit: Supplements S13 and S15), which is not uncommon for such modelling studies and shows that we do not yet fully understand the processes underlying alcohol-involved interpersonal violence. Finally, we applied logit transformation for proportions in meta-analytical methods, which does not account for a variance squeezing effect for very small (or large) proportions, resulting in large inverse variance weights for such estimates. Given that the observed prevalence was small in most studies, we believe that this effect did only marginally impact our results.

In this systematic review and modelling study, we provide first estimates on interpersonal violence from others’ drinking in the GBD regions of High Income and Central Europe, Eastern Europe, & Central Asia. According to our modelling, every fourth and every sixth person within these regions, respectively, experienced emotional violence from others’ drinking in 2019, constituting the most prevalent form of interpersonal violence from others’ drinking. This harm that extends beyond the alcohol user needs to be considered in the alcohol policy debate, as it has been established for tobacco control.^84^ Moreover, in light of apparent sex/gender differences and the contribution of men’s alcohol consumption to interpersonal violence from others’ drinking as observed in our models, sex/gender needs to be considered in the development of violence prevention strategies, including the development of more equitable alcohol policies accounting for the significant role of masculinity in alcohol-related violence.^85^ While alcohol control policies can result in meaningful reductions of violence-related crimes, they might not be enough to tackle interpersonal violence in the long term and should be complemented by individual- and family-level interventions.^12^

## Contributors

Conceptualisation: CK; data curation: CK, SK; formal analysis: CK; methodology: CK, CP, JM; project administration: CK; supervision: CP; validation: SK; visualisation: CK; writing – original draft: CK, JM, JR, TH; and writing – review & editing: all authors.

## Data sharing statement

The data file with the information extracted from the individual reports included in this modelling study, as well as all R scripts that support the findings of this review are openly available at the Figshare repository (DOI for R scripts: 10.6084/m9.figshare.25282294, for data files: 10.6084/m9.figshare.25282330).

## Editor note

The Lancet Group takes a neutral position with respect to territorial claims in published maps and institutional affiliations.

## Declaration of interests

JM worked as consultant for non-profit public health organisations. All other authors have no conflict to declare.
