# Supplementary material for: National and regional prevalence of interpersonal violence from others’ alcohol use: a systematic review and modelling study
Source: Lancet Reg Health Eur. 2024 Apr 17;40:100905. doi: 10.1016/j.lanepe.2024.100905 (PMC11047785; doi:10.1016/j.lanepe.2024.100905)
Supplement: Supplements S1–S18 [file mmc1.docx]

# Supplementary Material

**Title:** National and regional prevalence of interpersonal violence from others’ alcohol use: a systematic review and modelling study

**Authors:** Carolin Kilian, Sinja Klinger, Jakob Manthey, Jürgen Rehm, Taisia Huckle, Charlotte Probst

Table of content

[S1 – Detailed overview of the indicators considered in each category of violence 2](#_Toc161335880)

[S2 – PRISMA checklist tables 3](#_Toc161335881)

[S3 – Amendments from the protocol (Prospero) 7](#_Toc161335882)

[S4 – Search terms for databases 8](#_Toc161335883)

[S5 – Grey literature search 9](#_Toc161335884)

[S6 – Study selection 10](#_Toc161335885)

[S7 – Risk of bias assessment 11](#_Toc161335886)

[S8 – Study variables and covariates for modelling 12](#_Toc161335887)

[S9 – Global Burden of Disease study’s super region 13](#_Toc161335888)

[S10 – Investigation of secular trends 14](#_Toc161335889)

[S11 – Investigation of the COVID impact 15](#_Toc161335890)

[S12 – Final multivariate models used for prediction 16](#_Toc161335891)

[S13 – Model fit: physical violence from others’ drinking 17](#_Toc161335892)

[S14 – Observed prevalence of physical violence from others’ drinking in regions not modelled 18](#_Toc161335893)

[S15 – Model fit: emotional violence from others’ drinking 19](#_Toc161335894)

[S16 – Observed prevalence of emotional violence from others’ drinking in regions not modelled 20](#_Toc161335895)

[S17 – Sexual violence from others’ drinking: results from meta-regression models 21](#_Toc161335896)

[S18 – Intimate partner violence from the partner’s alcohol use: results from meta-regression models 22](#_Toc161335897)

[References 23](#_Toc161335898)

## S1 – Detailed overview of the indicators considered in each category of violence

**Table S1. Indicators used for different forms of violence**

| **Forms of violence** | **Indicators** |
| --- | --- |
| Physical violence | - been physically hurt due to them assaulting me or acting violently - being hit, pushed or shoved - having been threatened |
| Emotional violence | - been emotionally hurt or neglected - felt physically threatened or having been threatened - called names or otherwise insulted - have been harassed or bothered on the street or another public place or at home or another private setting - have been harassed, insulted or humiliated on the street or another public place or at home or another private setting - Had a serious argument that did NOT include physical violence - serious argument (not further specified) - Felt genuinely concerned that they may cause harm to my children or someone else’s children - felt threatened or afraid because of someone's drinking at home or another private setting |
| Sexual violence | - felt forced or pressured into sex or something sexual - unwanted sexual attention or someone behaved in a sexually inappropriate way - sexual harassment |

## S2 – PRISMA checklist tables

**Table S2. PRISMA 2020 checklist**

| **Section and Topic** | **Item #** | **Checklist item** | **Location where item is reported** |
| --- | --- | --- | --- |
| **TITLE** | | |  |
| Title | 1 | Identify the report as a systematic review. | M1 |
| **ABSTRACT** | | |  |
| Abstract | 2 | See the PRISMA 2020 for Abstracts checklist. | S2 |
| **INTRODUCTION** | | |  |
| Rationale | 3 | Describe the rationale for the review in the context of existing knowledge. | M4 |
| Objectives | 4 | Provide an explicit statement of the objective(s) or question(s) the review addresses. | M4 |
| **METHODS** | | |  |
| Eligibility criteria | 5 | Specify the inclusion and exclusion criteria for the review and how studies were grouped for the syntheses. | M5, S5 |
| Information sources | 6 | Specify all databases, registers, websites, organisations, reference lists and other sources searched or consulted to identify studies. Specify the date when each source was last searched or consulted. | M5 |
| Search strategy | 7 | Present the full search strategies for all databases, registers and websites, including any filters and limits used. | S4-S6 |
| Selection process | 8 | Specify the methods used to decide whether a study met the inclusion criteria of the review, including how many reviewers screened each record and each report retrieved, whether they worked independently, and if applicable, details of automation tools used in the process. | M5 |
| Data collection process | 9 | Specify the methods used to collect data from reports, including how many reviewers collected data from each report, whether they worked independently, any processes for obtaining or confirming data from study investigators, and if applicable, details of automation tools used in the process. | M5 |
| Data items | 10a | List and define all outcomes for which data were sought. Specify whether all results that were compatible with each outcome domain in each study were sought (e.g. for all measures, time points, analyses), and if not, the methods used to decide which results to collect. | M5-6 |
|  | 10b | List and define all other variables for which data were sought (e.g. participant and intervention characteristics, funding sources). Describe any assumptions made about any missing or unclear information. | S8 |
| Study risk of bias assessment | 11 | Specify the methods used to assess risk of bias in the included studies, including details of the tool(s) used, how many reviewers assessed each study and whether they worked independently, and if applicable, details of automation tools used in the process. | M5, S7 |
| Effect measures | 12 | Specify for each outcome the effect measure(s) (e.g. risk ratio, mean difference) used in the synthesis or presentation of results. | M5-7 |
| Synthesis methods | 13a | Describe the processes used to decide which studies were eligible for each synthesis (e.g. tabulating the study intervention characteristics and comparing against the planned groups for each synthesis (item #5)). | M6 |
|  | 13b | Describe any methods required to prepare the data for presentation or synthesis, such as handling of missing summary statistics, or data conversions. | M5-6 |
|  | 13c | Describe any methods used to tabulate or visually display results of individual studies and syntheses. | M6-7 |
|  | 13d | Describe any methods used to synthesize results and provide a rationale for the choice(s). If meta-analysis was performed, describe the model(s), method(s) to identify the presence and extent of statistical heterogeneity, and software package(s) used. | M6-7 |
|  | 13e | Describe any methods used to explore possible causes of heterogeneity among study results (e.g. subgroup analysis, meta-regression). | M7 |
|  | 13f | Describe any sensitivity analyses conducted to assess robustness of the synthesized results. | M7 |
| Reporting bias assessment | 14 | Describe any methods used to assess risk of bias due to missing results in a synthesis (arising from reporting biases). | M7 |
| Certainty assessment | 15 | Describe any methods used to assess certainty (or confidence) in the body of evidence for an outcome. | M7 |
| **RESULTS** | | |  |
| Study selection | 16a | Describe the results of the search and selection process, from the number of records identified in the search to the number of studies included in the review, ideally using a flow diagram. | M8 |
|  | 16b | Cite studies that might appear to meet the inclusion criteria, but which were excluded, and explain why they were excluded. | Table 1 |
| Study characteristics | 17 | Cite each included study and present its characteristics. | Table 1, S11 |
| Risk of bias in studies | 18 | Present assessments of risk of bias for each included study. | Table 1 |
| Results of individual studies | 19 | For all outcomes, present, for each study: (a) summary statistics for each group (where appropriate) and (b) an effect estimate and its precision (e.g. confidence/credible interval), ideally using structured tables or plots. | Table 1, S14, S16, full data available online |
| Results of syntheses | 20a | For each synthesis, briefly summarise the characteristics and risk of bias among contributing studies. | M8, Table 1 |
|  | 20b | Present results of all statistical syntheses conducted. If meta-analysis was done, present for each the summary estimate and its precision (e.g. confidence/credible interval) and measures of statistical heterogeneity. If comparing groups, describe the direction of the effect. | M8-M10, S12 |
|  | 20c | Present results of all investigations of possible causes of heterogeneity among study results. | S17, S18 |
|  | 20d | Present results of all sensitivity analyses conducted to assess the robustness of the synthesized results. | S17, S18 |
| Reporting biases | 21 | Present assessments of risk of bias due to missing results (arising from reporting biases) for each synthesis assessed. | S17, S18 |
| Certainty of evidence | 22 | Present assessments of certainty (or confidence) in the body of evidence for each outcome assessed. | Table 2, Figures 5-6 |
| **DISCUSSION** | | |  |
| Discussion | 23a | Provide a general interpretation of the results in the context of other evidence. | M10 |
|  | 23b | Discuss any limitations of the evidence included in the review. | M11 |
|  | 23c | Discuss any limitations of the review processes used. | M11 |
|  | 23d | Discuss implications of the results for practice, policy, and future research. | M12 |
| **OTHER INFORMATION** | | |  |
| Registration and protocol | 24a | Provide registration information for the review, including register name and registration number, or state that the review was not registered. | M3 |
|  | 24b | Indicate where the review protocol can be accessed, or state that a protocol was not prepared. | M3 |
|  | 24c | Describe and explain any amendments to information provided at registration or in the protocol. | S3 |
| Support | 25 | Describe sources of financial or non-financial support for the review, and the role of the funders or sponsors in the review. | M13 |
| Competing interests | 26 | Declare any competing interests of review authors. | M13 |
| Availability of data, code and other materials | 27 | Report which of the following are publicly available and where they can be found: template data collection forms; data extracted from included studies; data used for all analyses; analytic code; any other materials used in the review. | M13 |

M: manuscript, S: supplement. *From:* Page MJ, McKenzie JE, Bossuyt PM, Boutron I, Hoffmann TC, Mulrow CD, et al. The PRISMA 2020 statement: an updated guideline for reporting systematic reviews. BMJ 2021;372:n71. doi: 10.1136/bmj.n71

**Table S3. PRISMA 2020 for Abstracts checklist**

| **Section and Topic** | **Item #** | **Checklist item** | **Reported (Yes/No)** |
| --- | --- | --- | --- |
| **TITLE** | | |  |
| Title | 1 | Identify the report as a systematic review. | Yes |
| **BACKGROUND** | | |  |
| Objectives | 2 | Provide an explicit statement of the main objective(s) or question(s) the review addresses. | Yes |
| **METHODS** | | |  |
| Eligibility criteria | 3 | Specify the inclusion and exclusion criteria for the review. | Yes |
| Information sources | 4 | Specify the information sources (e.g. databases, registers) used to identify studies and the date when each was last searched. | Yes |
| Risk of bias | 5 | Specify the methods used to assess risk of bias in the included studies. | Yes |
| Synthesis of results | 6 | Specify the methods used to present and synthesise results. | Yes |
| **RESULTS** | | |  |
| Included studies | 7 | Give the total number of included studies and participants and summarise relevant characteristics of studies. | Yes |
| Synthesis of results | 8 | Present results for main outcomes, preferably indicating the number of included studies and participants for each. If meta-analysis was done, report the summary estimate and confidence/credible interval. If comparing groups, indicate the direction of the effect (i.e. which group is favoured). | Yes |
| **DISCUSSION** | | |  |
| Limitations of evidence | 9 | Provide a brief summary of the limitations of the evidence included in the review (e.g. study risk of bias, inconsistency and imprecision). | n/a |
| Interpretation | 10 | Provide a general interpretation of the results and important implications. | Yes |
| **OTHER** | | |  |
| Funding | 11 | Specify the primary source of funding for the review. | n/a |
| Registration | 12 | Provide the register name and registration number. | Yes |

N/A: not applicable. *From:* Page MJ, McKenzie JE, Bossuyt PM, Boutron I, Hoffmann TC, Mulrow CD, et al. The PRISMA 2020 statement: an updated guideline for reporting systematic reviews. BMJ 2021;372:n71. doi: 10.1136/bmj.n71

## S3 – Amendments from the protocol (Prospero)

**Title and review question**

The preregistered research questions aimed at estimating the national, regional, and global prevalence of interpersonal violence from others’ drinking. However, we omitted the estimation of the global prevalence, as several GBD super regions were covered insufficiently.

**Statistical analyses**

In the preregistration, we have proposed to include the prevalence of interpersonal violence as predictor in the fractional response regression models. After careful deliberations, we have decided not to include this variable given its theoretical correlation with our outcome (i.e., interpersonal violence from others’ drinking). We further intended to use regions of the World Health Organization first, but eventually decided to use the regional grouping of the Global Burden of Disease given the geographical locations of countries in the identified reports.

## S4 – Search terms for databases

Ovid SP

(((interpersonal OR physical OR sexual OR psychological OR verbal OR emotional OR “intimate partner” OR domestic) AND (violen* OR assault? OR abuse OR harm)) OR “physical attack” OR fight OR rape OR “harm from other*” OR “second?hand harm”).ti,ab.

AND (alcohol OR dr#nk* OR ethanol).ti,ab.

AND (frequency OR prevalence).ti,ab.

AND (women OR men OR female OR male OR adult? OR “general populat*”).ti,ab.

Web of Science

TS=(((interpersonal OR physical OR sexual OR psychological OR verbal OR emotional OR intimate partner OR domestic) AND (violen* OR assault$ OR abuse OR harm)) OR physical attack OR fight OR rape OR harm from other* OR second$hand harm)

AND TS=(alcohol* OR drink* OR drunk OR drank OR ethanol)

AND TS= (frequency OR prevalence)

AND TS=(women OR men OR female OR male OR adult$ OR general populat*)

PubMed

(((“interpersonal”[Title/Abstract] OR “physical”[Title/Abstract] OR “sexual”[Title/Abstract] OR “psychological”[Title/Abstract] OR “verbal”[Title/Abstract] OR “emotional”[Title/Abstract] OR “intimate partner”[Title/Abstract] OR “domestic”[Title/Abstract]) AND (“violen*”[Title/Abstract] OR “assault#”[Title/Abstract] OR “abuse”[Title/Abstract] OR “harm”[Title/Abstract])) OR “physical attack”[Title/Abstract] OR “fight”[Title/Abstract] OR “rape”[Title/Abstract] OR “harm from other*”[Title/Abstract] OR “second#hand harm”[Title/Abstract]))

AND (“alcohol”[Title/Abstract] OR “dr#nk*”[Title/Abstract] OR “ethanol”[Title/Abstract])

AND (“frequency”[Title/Abstract] OR “prevalence”[Title/Abstract])

AND (“women”[Title/Abstract] OR “men”[Title/Abstract] OR “female”[Title/Abstract] OR “male”[Title/Abstract] OR “adult#”[Title/Abstract] OR “general populat*”[Title/Abstract])

## S5 – Grey literature search

Documentation of Google (Scholar) Search

For all regions and sub-regions, the online databases Google and Google Scholar will be searched using the search terms listed below. The first two result pages of each search will be screened for potentially relevant literature that is any publication related to alcohol or substance use and interpersonal violence. All hints on possible surveys will be followed up using Google. If review studies are identified, their reference lists are screened for eligible primary research reports. Additionally, the web pages of the World Health Organization (including regional offices), the Organization for Economic Cooperation and Development (OECD) and the Institute for Health Metrics and Evaluation (IHME) will be checked for reports on alcohol’s harm to others.

The search will be performed in English. If needed, Google translate will be used for any literature written in a language other than English.

Search terms:

- Alcohol’s harm to others
- Harm from other’s drinking
- Substance use AND interpersonal violence
- Alcohol AND interpersonal violence
- Substance use AND violence
- Alcohol AND violence
- Substance use AND fight
- Alcohol AND fight

**Table S4. Search on articles and reports on alcohol’s harm to others in the following regions:**

| African Region | Africa, Sub-Saharan Africa, North Africa, Eastern Sub-Saharan, Western Sub-Saharan, Southern Sub-Saharan |
| --- | --- |
| South-East Asian Region | Asia, South Asia, East Asia, Southeast Asia, Western Asia, Central Asia |
| Eastern Mediterranean Region | Eastern Mediterranean, Middle East, Arabia, Maghreb |
| Western Pacific Region | Western Pacific, Oceania, Australasia, Melanesia, Micronesia, Polynesia |
| European Region | Eastern Europe, Western Europe, Central Europe, Northern Europe, European Union, Baltic countries, Balkans, Mediterranean countries, Nordic countries / Scandinavia |
| The Americas | North America, South America, Central America, Latin America, Caribbean |
| Income classes | High income, Upper middle income, Lower middle income, Low income |

Inclusion Criteria

Studies meeting the following criteria will be included: (1) general population studies of mainly adults, (2) assessing interpersonal violence from other’s drinking (including physical, emotional/verbal, and sexual violence), and (3) reporting its prevalence either by gender or in the total sample. Studies that collected data only from specific regions within countries, such as rural areas or cities will also be included as long as the target population is well-described.

Results

Any studies or reports of interest will be noted in a separate Excel sheet. The ones available will be marked green, the ones not available online will be marked red and possibly searched for by consulting national experts.

## S6 – Study selection

**Table S5. Inclusion and exclusion criteria for study selection**

| **Criterion** | **Inclusion** | **Exclusion** |
| --- | --- | --- |
| Outcome | Prevalence of interpersonal violence from other’s drinking (as defined above), reported either in the overall sample or by sex/gender | Any harm from other’s drinking that is not interpersonal violence as defined in this study (e.g., financial harm)  Interpersonal violence not related to other’s drinking |
| Method | Assessment of interpersonal violence from other’s drinking as defined above | . |
| Design | Original quantitative research reports | Qualitative reports  Expert or opinion letters |
| Sample | Samples reflecting the general adult population;  for surveys, samples have to be drawn either by means of  (a) probabilistic sampling or  (b) non-probabilistic sampling accounting for sampling bias by, for example, the use of sample weights | Samples that do not reflect the general adult population by covering, for example, a selective group of persons, such as college students or only homeless people |
| Results | Prevalence of interpersonal violence from other’s drinking reported in %  Or sufficient details to calculate the prevalence | . |
| Language | No restrictions | . |
| Time | No restrictions | . |

## S7 – Risk of bias assessment

The scale was adopted for the purpose of this research and contained two items in each of three categories (selection, comparability, and outcome). Items were scored 1 point if the relevant criterion was satisfied, so that each category was awarded a maximum of 2 points, with a higher score indicating a lower ROB. A study’s overall ROB was then based on the category with the lowest score, distinguishing between low (2 points in 2 categories, 1 point in 1 category), moderate (1 point in two to three categories, no category with 0 points), and critical ROB (at least one study with 0 points).

**Table S6. Adapted version of the Newcastle-Ottawa Quality Assessment Scale for cross-sectional studies**

| **Selection (up to 2 points)** | |
| --- | --- |
| **#1 Representativeness of the sample** | 1. truly representative of the average adult general population in the community (1 point) 2. somewhat representative of the average adult general population in the community (1 point) 3. selected group of users, e.g., nurses, volunteers (0 point) 4. no description of the derivation of the cohort (0 point) |
| **#2 *Non-respondents*** | 1. Comparability between respondents and non-respondents characteristics is established, and the response rate is satisfactory (1 point) *– response rate > 70%* 2. The response rate is unsatisfactory, or the comparability between respondents and nonrespondents is unsatisfactory. (0 point) 3. No description of the response rate or the characteristics of the responders and the non-responders. (0 point) |
| **#3 *Ascertainment of the exposure*** | *Not relevant* |
| **Comparability (up to 2 points)** | |
| **#4 Study controls for sex/*gender* *by presenting prevalence separately for women and men*** | 1. Yes (1 point) 2. No (0 point) |
| **#5 Study controls for additional factors, *i.e., the relationship between the victim and aggressor*** | 1. Yes (1 point) 2. No (0 point) |
| **Outcome (up to 2 points)** | |
| **#6 Assessment of outcome** | 1. Independent blind assessment (1 point) 2. Record linkage (1 point) 3. Self-report (0 point) 4. No description (0 point) |
| **#7 *Sample weights were applied for estimating prevalence*** | 1. Yes (1 point) 2. No (0 point) |

Changes highlighted in italics.

## S8 – Study variables and covariates for modelling

**Table S7. Overview of variables assessed for their unique association with the observed prevalence of interpersonal violence from others’ drinking.**

| **Variable** | **Explanation/source** |
| --- | --- |
| Regional identifier | Global Burden of Disease (GBD) study’s super region:^1^ Central Europe, Eastern Europe, & Central Asia; High Income, Latin America & Caribbean; North Africa & Middle East; South Asia; South-East Asia, East Asia, & Oceania; Sub-Saharan Africa |
| **Study variables** | |
| Study sample (sex/gender) | Categorial: men, women, vs. total sample (men and women) |
| Reference period | Binary: past 12 months vs. lifetime prevalence |
| Sampling | Binary: probabilistic vs. non-probabilistic sampling or not specified |
| Sampling weights | Binary: study used sampling weights vs. did not use sampling weights or prevalence was self-calculated |
| Role of survey respondent | Binary: victim vs. aggressor |
| Risk of bias | Binary: critical vs. moderate/low |
| **Covariates** | |
| Alcohol use^†^ | - Prevalence lifetime abstainers (15+) - Total per capita consumption in liters pure alcohol (15+) - Total per capita consumption in liters pure alcohol among current alcohol users only (15+) - Total per capita consumption in liters pure alcohol among men only (15+) - Prevalence heavy episodic drinking (i.e., at least one occasion with ≥ 60g pure alcohol in the past 30 days; 15+) - Combined indicator of total per capita alcohol consumption and the prevalence of heavy episodic drinking (15+)   Source: ^2^ |
| Economic wealth^‡§^ | Per capita gross domestic product purchasing power parity in international dollar, source: ^3^ |
| Income inequality^‡§*^ | Gini index, source: ^4^ (for New Zealand: ^5^, for Hong Kong: ^6^) |
| Unemployment^‡§^ | Proportion of total labour force unemployed, source: ^7^ |
| Male population | Proportion of males within the total population aged 15 to 64 years, source: ^8^ |
| Young population | Proportion of 15-to-24-year-olds within the total population aged 15 to 64 years, source: ^8^ |

† data of 2019 was used for years ≥ 2020. ‡ missing data was estimated using simple linear imputation. § No data was available for Andorra (High Income region). * Data was missing for Cambodia and estimated as the mean of Thailand, Laos, and Vietnam; no data was available for Brunei Darussalam and Singapore (High Income region).

## S9 – Global Burden of Disease study’s super region

**Central Europe, Eastern Europe, & Central Asia:**

Albania, Armenia, Azerbaijan, Belarus, Bosnia and Herzegovina, Croatia, Czech Republic, Estonia, Georgia, Hungary, Kazakhstan, Kyrgyzstan, Latvia, Lithuania, Macedonia, Moldova, Mongolia, Montenegro, Poland, Romania, Russia, Serbia, Slovakia, Slovenia, Tajikistan, Turkmenistan, Ukraine, Uzbekistan

**High Income:**

Andorra, Argentina, Australia, Austria, Belgium, Brunei Darussalam, Canada, Chile, Cyprus, Denmark, Finland, France, Germany, Greece, Greenland, Iceland, Ireland, Israel, Italy, Japan, Luxembourg, Malta, Netherlands, New Zealand, Norway, Portugal, Singapore, South Korea, Spain, Sweden, Switzerland, United Kingdom, United States, Uruguay

**Latin America & Caribbean:**

Antigua and Barbuda, Barbados, Belize, Bermuda, Bolivia, Brazil, Colombia, Costa Rica, Cuba, Dominica, Dominican Republic, Ecuador, El Salvador, Grenada, Guatemala, Guyana, Haiti, Honduras, Jamaica, Mexico, Nicaragua, Panama, Paraguay, Peru, Puerto Rico, Saint Lucia, Saint Vincent and the Grenadines, Suriname, The Bahamas, Trinidad and Tobago, Venezuela

**North Africa & Middle East:**

Afghanistan, Algeria, Bahrain, Egypt, Iran, Iraq, Jordan, Kuwait, Lebanon, Libya, Morocco, Oman, Palestine, Qatar, Saudi Arabia, Sudan, Syria, Tunisia, Turkey, United Arab Emirates, Yemen

**South Asia:**

Bangladesh, Bhutan, India, Nepal, Pakistan

**Sub-Saharan Africa:**

Angola, Benin, Botswana, Burkina Faso, Burundi, Cameroon, Cape Verde, Central African Republic, Chad, Comoros, Congo, Cote d'Ivoire, Democratic Republic of the Congo, Djibouti, Equatorial Guinea, Eritrea, Ethiopia, Gabon, Ghana, Guinea, Guinea‐Bissau, Kenya, Lesotho, Liberia, Madagascar, Malawi, Mali, Mauritania, Mozambique, Namibia, Niger, Nigeria, Rwanda, Sao Tome and Principe, Senegal, Sierra Leone, Somalia, South Africa, South Sudan, Swaziland, Tanzania, The Gambia, Togo, Uganda, Zambia, Zimbabwe

**Southeast Asia:**

American Samoa, Cambodia, China, Federated States of Micronesia, Fiji, Guam, Indonesia, Kiribati, Laos, Malaysia, Maldives, Marshall Islands, Mauritius, Myanmar, North Korea, Papua New Guinea, Philippines, Samoa, Seychelles, Solomon Islands, Sri Lanka, Taiwan, Thailand, Timor‐Leste, Tonga, Vanuatu, Vietnam

## S10 – Investigation of secular trends


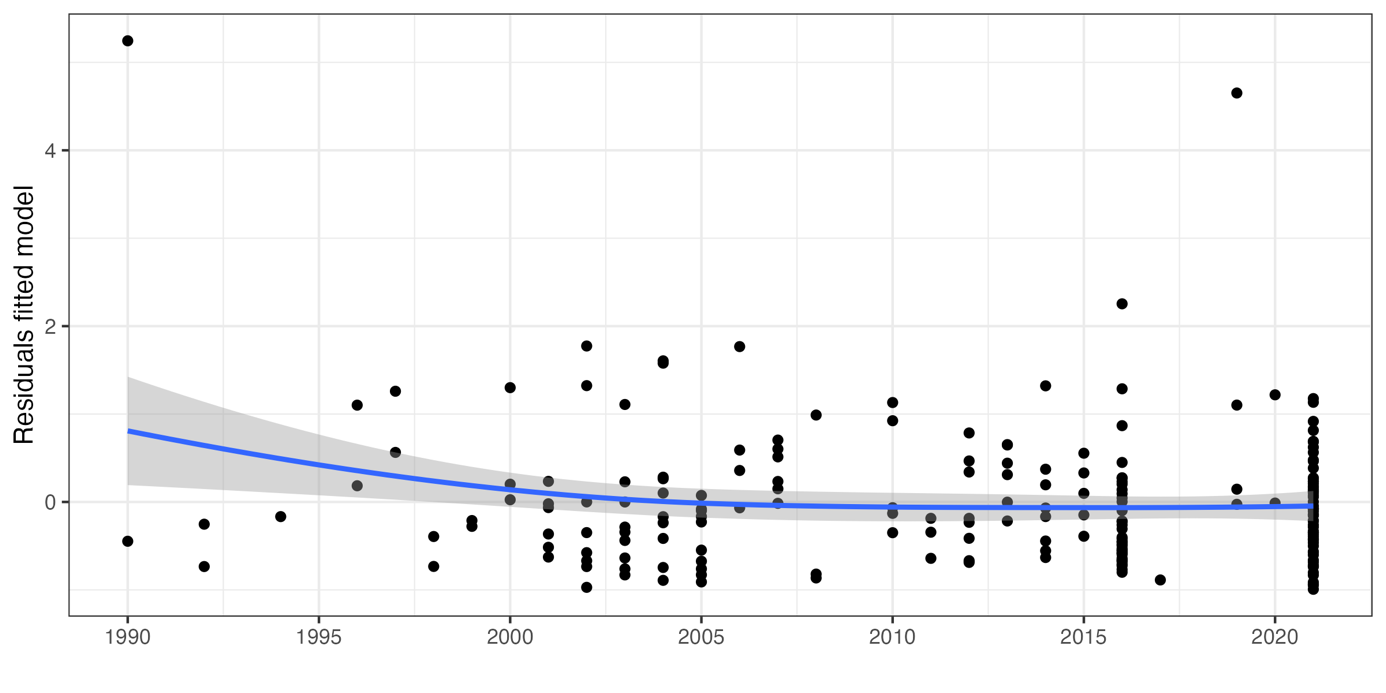


**Figure S1. Secular trend of the residuals of the fitted model for the prevalence of physical violence from other's drinking.**


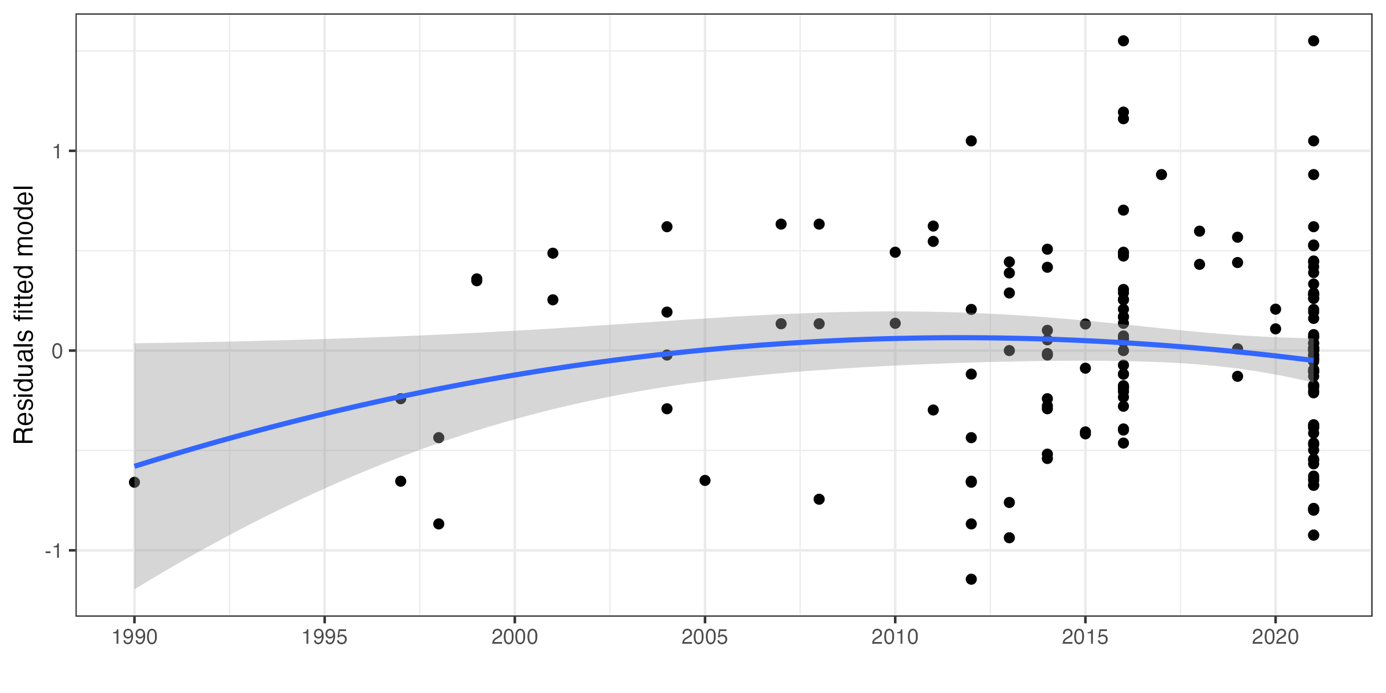


**Figure S2. Secular trend of the residuals of the fitted model for the prevalence of emotional violence from other's drinking.**

## S11 – Investigation of the COVID impact

We have used a subsample of studies from countries, where at least two measurements were available, to investigate the impact of the COVID-19 pandemic on the prevalence of interpersonal violence from other’s drinking. For each country included the subsample at least one measurement before and one during or after 2020 were required to be available. Data were analysed using multi-level fractional regression models (glmmPQL function with quasibinomial logit link, R package *MASS*, version 7.3.57)^9^ with country being the random intercept. The outcome variable was the prevalence of (a) physical or (b) emotional violence from other’s drinking. The year of assessment served as dummy-coded independent variable (assessment before 2020 vs. assessment in 2020 or later). Additional covariates (i.e., survey sample, reference period, sampling, role of survey respondent, risk of bias, GBD regions) were investigated and retained in the model, if significantly associated with the outcome. All models were weighted using each study’s inverse variance weight extracted from a random-effects meta-analysis applying a logit transformation for proportions and using the DerSimonian-Laird estimator.

**Table S8. Results of the multi-level fractional response regression model: prevalence of physical violence from other’s drinking (logit-transformed).**

| **Variable** | **Physical violence from other’s drinking** | | |
| --- | --- | --- | --- |
|  | **Coefficient** | **SE** | **p-value** |
| COVID: assessment in 2020 or later (ref.: no): yes | 0·09 | 0·16 | ·594 |
| Survey sample (ref.: men): women | -0·37 | 0·08 | < ·001 |
| Intercept | -3·25 | 0·17 | < ·001 |

Number of observations: 104, number of countries: 33. SE: standard error.

**Table S9. Results of the multi-level fractional response regression model: prevalence of emotional violence from other’s drinking (logit-transformed).**

| **Variable** | **Emotional violence from other’s drinking** | | |
| --- | --- | --- | --- |
|  | **Coefficient** | **SE** | **p-value** |
| COVID: assessment in 2020 or later (ref.: no): yes | 0·29 | 0·19 | ·129 |
| Sampling (ref: non-probabilistic sample): probabilistic sample | 0·55 | 0·15 | ·001 |
| GBD region (ref.: Central Europe, Eastern Europe, and Central Asia): High-Income Countries | -0·51 | 0·14 | ·001 |
| Intercept | -1·85 | 0·21 | < ·001 |

Number of observations: 102, number of countries: 33. SE: standard error.

## S12 – Final multivariate models used for prediction

**Table S10. Results of the final multivariate fractional response model used for estimating the country- and sex/gender-specific prevalence of physical violence from others’ drinking (logit-transformed).**

| **Variable** | | **Physical violence from others’ drinking** | | |
| --- | --- | --- | --- | --- |
|  |  | **Coefficient** | **SE** | **p-value** |
| Survey sample (ref.: men) | |  |  |  |
| Men and women combined | -0·51 | 0·18 | ·005 |  |
| Women | -0·52 | 0·10 | < ·001 |  |
| Survey reference period (ref.: lifetime) | |  |  |  |
| Past 12 months | -1·03 | 0·25 | < ·001 |  |
| Proportion of male population | | -15·97 | 7·87 | ·044 |
| Per capita alcohol consumption among men | | -0·02 | 0·02 | ·515 |
| Per capita GDP PPP | | 0·0000006 | 0·0000033 | ·854 |
| GBD region (ref.: High-Income Countries) | |  |  |  |
| Central Europe, Eastern Europe, and Central Asia | -2·13 | 0·72 | ·004 |  |
| Latin America and Caribbean | 2·71 | 1·20 | ·025 |  |
| South Asia | 2181·00 | 624·50 | ·001 |  |
| South-East Asia, East Asia, and Oceania | 37·86 | 16·89 | ·026 |  |
| Proportion of male population*South Asia | | -4208·00 | 1206·00 | ·001 |
| Proportion of male population*South-East Asia, East Asia, and Oceania | | -71·55 | 33·45 | ·034 |
| Per capita alcohol consumption among men*Central Europe, Eastern Europe, and Central Asia | | 0·11 | 0·04 | ·003 |
| Per capita alcohol consumption among men*Latin America and Caribbean | | -0·24 | 0·11 | ·034 |
| Per capita GDP PPP*South-East Asia, East Asia, and Oceania | | -0·0001657 | 0·0000529 | ·002 |
| Intercept | | 6·41 | 4·03 | ·113 |

Note. n = 217. R^2^ = 55·9%. GDP PPP: gross domestic product purchasing power parity. SE: standard error. Ref: reference.

**Table S11. Results of the final multivariate fractional response model used for estimating the country-specific prevalence of emotional violence from others’ drinking (logit-transformed).**

| **Variable** | | **Emotional violence from others’ drinking** | | |
| --- | --- | --- | --- | --- |
|  |  | **Coefficient** | **SE** | **p-value** |
| Sampling (ref.: non-probabilistic): probabilistic | | 0·45 | 0·11 | < ·001 |
| Per capita alcohol consumption among men | | 0·03 | 0·02 | ·068 |
| Gini index | | -0·01 | 0·02 | ·381 |
| Unemployment (z-standardised) | | 0·02 | 0·02 | ·368 |
| Male population | | -11·95 | 7·03 | ·092 |
| GBD region (ref.: High-Income Countries) | |  |  |  |
| Central Europe, Eastern Europe, and Central Asia | -0·47 | 0·75 | ·533 |  |
| Latin America and Caribbean | -0·84 | 0·85 | ·325 |  |
| South Asia | 1·56 | 0·31 | < ·001 |  |
| South-East Asia, East Asia, and Oceania | 5·27 | 1·44 | < ·001 |  |
| Gini index*Central Europe, Eastern Europe, and Central Asia | | 0·03 | 0·02 | ·274 |
| Gini index*South-East Asia, East Asia, and Oceania | | -0·12 | 0·04 | ·003 |
| Intercept | | 3·87 | 3·85 | ·316 |

Note. N = 164. R^2^ = 45·7%. SE: standard error. Ref: reference.

## S13 – Model fit: physical violence from others’ drinking


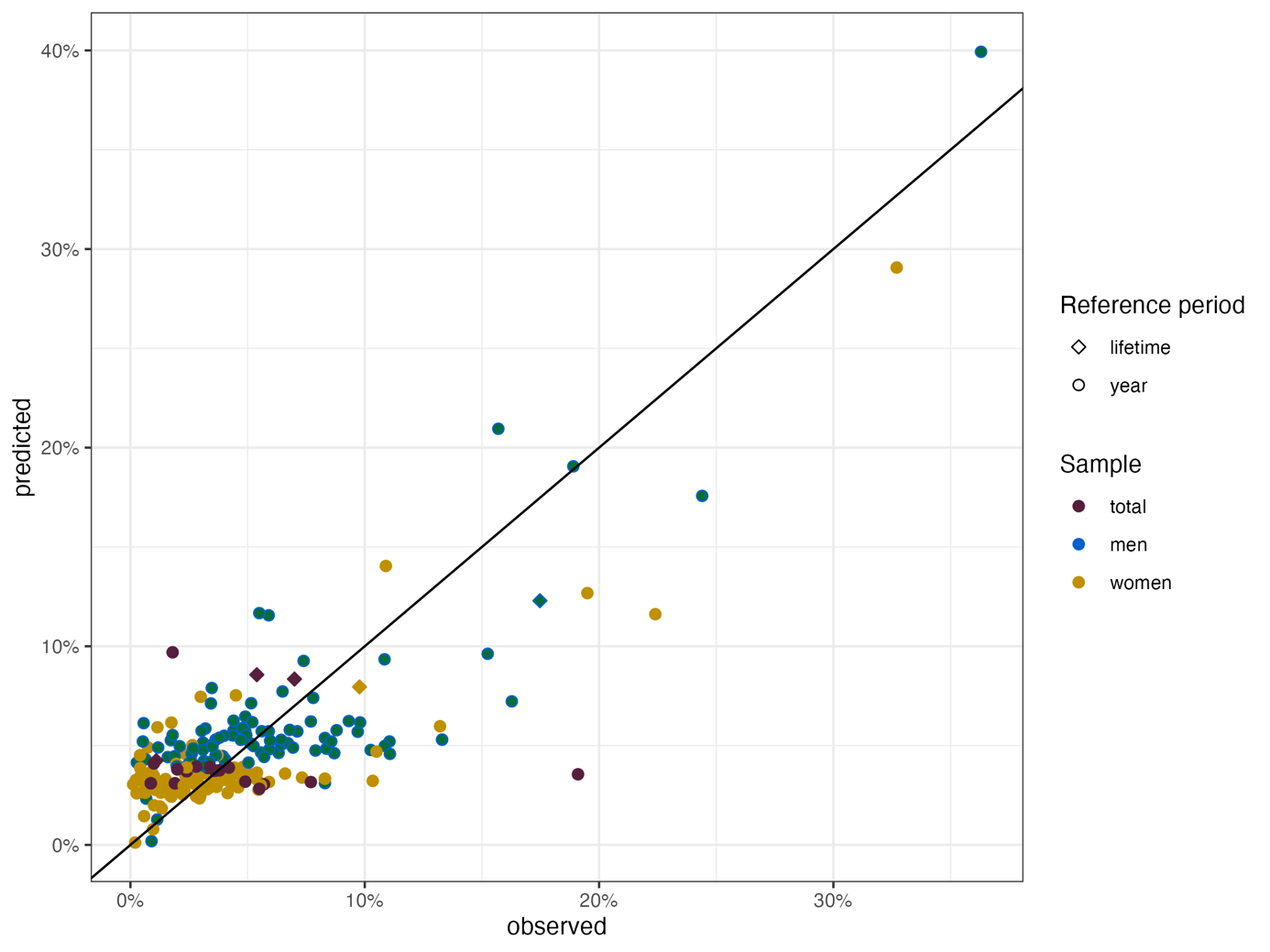


**Figure S5. Model performance: displayed are the observed (x-axis) and predicted (y-axis) prevalences of physical violence from others drinking.**

## S14 – Observed prevalence of physical violence from others’ drinking in regions not modelled

**Table S12. Prevalence of physical violence from others’ drinking as observed in countries of region not modelled.**

| **Reference** | **Country** | **Year** | **Physical violence from others’ drinking** | | |
| --- | --- | --- | --- | --- | --- |
|  |  |  | **Women (n)** | **Men (n)** | **Total (n)** |
| **Latin America and Caribbean** | | | | | |
| Graham et al. 2011 | Belize^‡^ | 2005 | 1·0 (1,907) | 6·6 (1,722) | . |
| Moreira et al. 2011 | Brazil | 2005 | . | . | 1·1 (454)* |
| Graham et al. 2011 | Brazil^‡^ | 2006 | 2·4 (1,052) | 11·4 (769) | . |
| Graham et al. 2011 | Costa Rica^‡^ | 2003 | 3·8 (778) | 20·7 (384) | . |
| Graham et al. 2011 | Nicaragua^‡^ | 2005 | 3·2 (1,378) | 12·3 (587) | . |
| Graham et al. 2011 | Peru^‡^ | 2005 | 0·8 (1,013) | 6·0 (516) | . |
| **South Asia** | | | | | |
| Graham et al. 2011 | India^‡^ | 2003 | . | 9·3 (1,327) | . |
| Waleewong et al. 2018 | India^‡^ | 2014 | 32·7 (1,616) | 36·3 (1,668) | . |
| **South-East Asia, East Asia, and Oceania** | | | | | |
| Yu et al. 2022 | Hong Kong SAR, China | 2019 | 0·2 (1,720) | 0·9 (1,480) | . |
| Waleewong et al. 2018 | Laos^‡^ | 2013 | 10·9 (602) | 15·7 (610) | . |
| Waleewong et al. 2018 | Sri Lanka^‡^ | 2014 | 22·4 (1,191) | 24·4 (1,095) | . |
| Waleewong et al. 2018 | Thailand^‡^ | 2012 | 4·5 (1,001) | 5·5 (694) |  |
| Hanh et al. 2019 | Vietnam^‡^ | 2017 | . | . | 1·8 (2,394) |
| Waleewong et al. 2018 | Vietnam ^‡^ | 2012 | 19·5 (735) | 18·9 (712) | . |
| **Sub-Saharan Africa** | | | | | |
| Graham et al. 2011 | Nigeria^‡^ | 2003 | 2·2 (948) | 2·0 (1,092) | . |

‡ Data was collected in metropolitan areas or in specific regions or states only. *Lifetime prevalence. If not otherwise specified, past-year point prevalence is shown.

## S15 – Model fit: emotional violence from others’ drinking


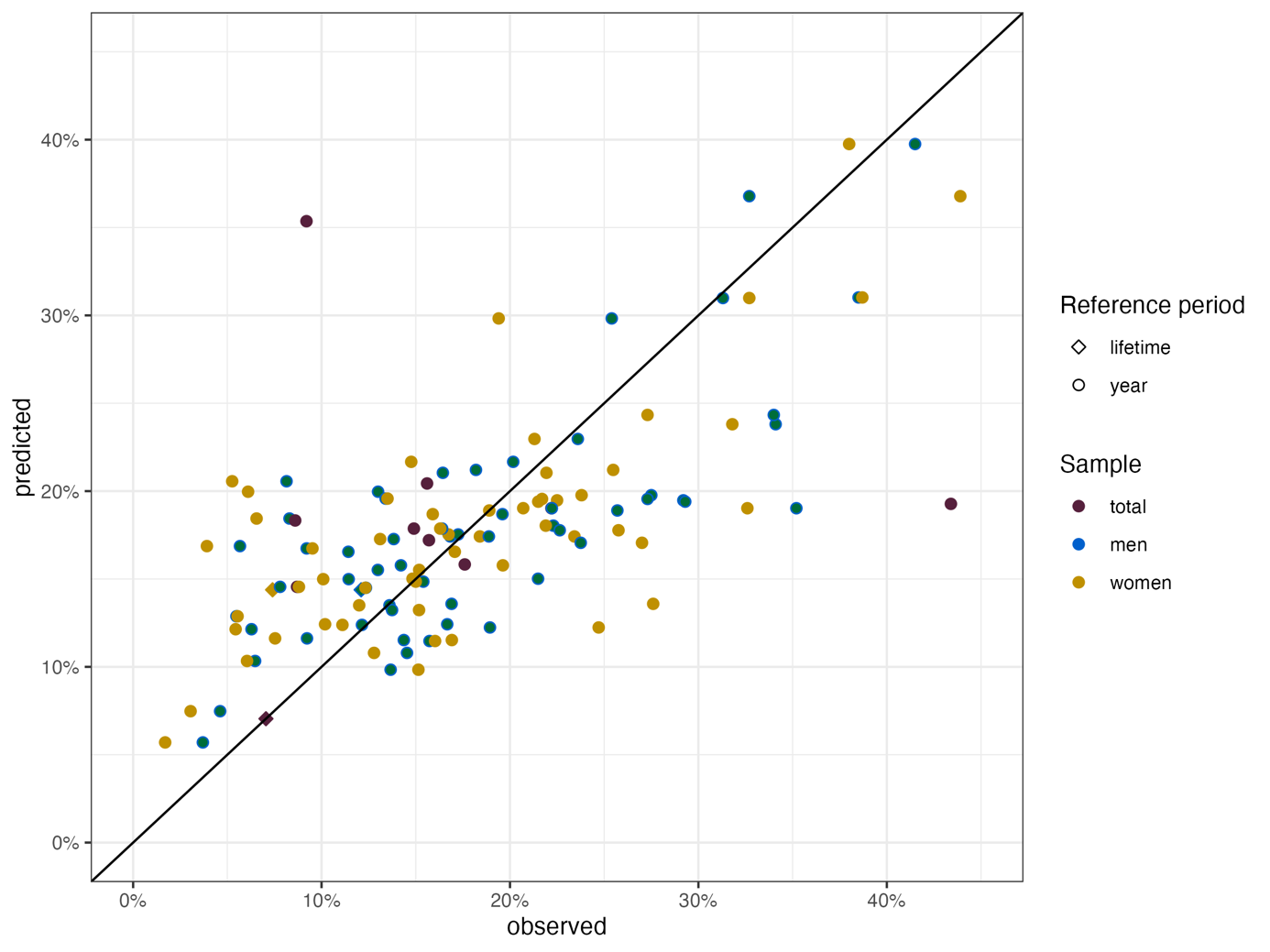


**Figure S6. Model performance: displayed are the observed (x-axis) and predicted (y-axis) prevalences of emotional violence from others drinking.**

## S16 – Observed prevalence of emotional violence from others’ drinking in regions not modelled

**Table S13. Prevalence of emotional violence from others’ drinking as observed in countries of region not modelled.**

| **Reference** | **Country** | **Year** | **Emotional violence from others’ drinking** | | |
| --- | --- | --- | --- | --- | --- |
|  |  |  | **Women (n)** | **Men (n)** | **Total (n)** |
| **Latin America and Caribbean** | | | | | |
| Moreira et al. 2011 | Brazil | 2005 | . | . | 7·0 (454)* |
| **South Asia** | | | | | |
| Waleewong et al. 2018 | India^‡^ | 2014 | 38·0 (1,616) | 41·5 (1,668) | . |
| **South-East Asia, East Asia, and Oceania** | | | | | |
| Yu et al. 2022 | Hong Kong SAR, China | 2019 | 1·7 (1,720) | 3·7 (1,480) | . |
| Waleewong et al. 2018 | Laos^‡^ | 2013 | 19·4 (602) | 25·4 (610) | . |
| Waleewong et al. 2018 | Sri Lanka^‡^ | 2014 | 31·8 (1,191) | 34·1 (1,095) | . |
| Waleewong et al. 2018 | Thailand^‡^ | 2012 | 27·3 (1,001) | 34·0 (694) |  |
| Hanh et al. 2019 | Vietnam^‡^ | 2017 | . | . | 9·2 (2,394) |
| Waleewong et al. 2018 | Vietnam ^‡^ | 2012 | 38·7 (735) | 38·5 (712) | . |

^‡^ Data was collected in metropolitan areas or in specific regions or states only. *Lifetime prevalence. If not otherwise specified, past-year point prevalence is shown.

## S17 – Sexual violence from others’ drinking: results from meta-regression models

**Table S14. Results from meta-regression models: sexual violence from others’ drinking (outcome).**

|  | **Estimate**  **(95% CI)** | **95% PI** | ***p*-value^‡^** | **Q (p-value)** | **I^2^** |
| --- | --- | --- | --- | --- | --- |
| **Main model without intercept (without intercept, k = 19)** | | | | | |
| Sample: men | 1·3 (0·5-3·3) | 0·1-16·9 | ref | 514·01 (< ·001) | 97·4 |
| Sample: men women combined | 0·9 (0·2-3·8) | 0·0-14·6 | ·639 | . | . |
| Sample: women | 3·4 (1·4-8·3) | 0·2-35·3 | <·001 | . | . |
| **Sensitivity analysis: accounting for risk of bias^b^ (without intercept, k = 19)** | | | | | |
| Sample: men | 1·0 (0·2-4·1) | 0·1-17·6 | ref | 490·37 (< ·001) | 97·6 |
| Sample: men women combined | 0·6 (0·1-4·4) | 0·0-15·2 | ·641 | . | . |
| Sample: women | 2·6 (0·6-10·3) | 0·1-36·3 | <·001 | . | . |
| **Sensitivity analysis: main model excluding Gell et al. 2015 (without intercept, k = 17)** | | | | | |
| Sample: men | 1·0 (0·4-2·5) | 0·1-11·4 | ref | 408·42 (< ·001) | 95·7 |
| Sample: men women combined | 0·9 (0·2-3·4) | 0·1-12·3 | ·837 | . | . |
| Sample: women | 2·4 (1·0-6·1) | 0·2-24·9 | <·001 | . | . |

Note: CI = confidence interval. PI = prediction interval (estimated for models without intercept only). Ref = reference. *I*^2^ indicates between-study heterogeneity. Three-level model to account for clustering of data within individual studies, using study ID as cluster.^10^ ^‡^ significance of the comparison of each category with reference. ^b^ no significant difference between studies with low/moderate versus critical risk of bias, *p* = ·621.


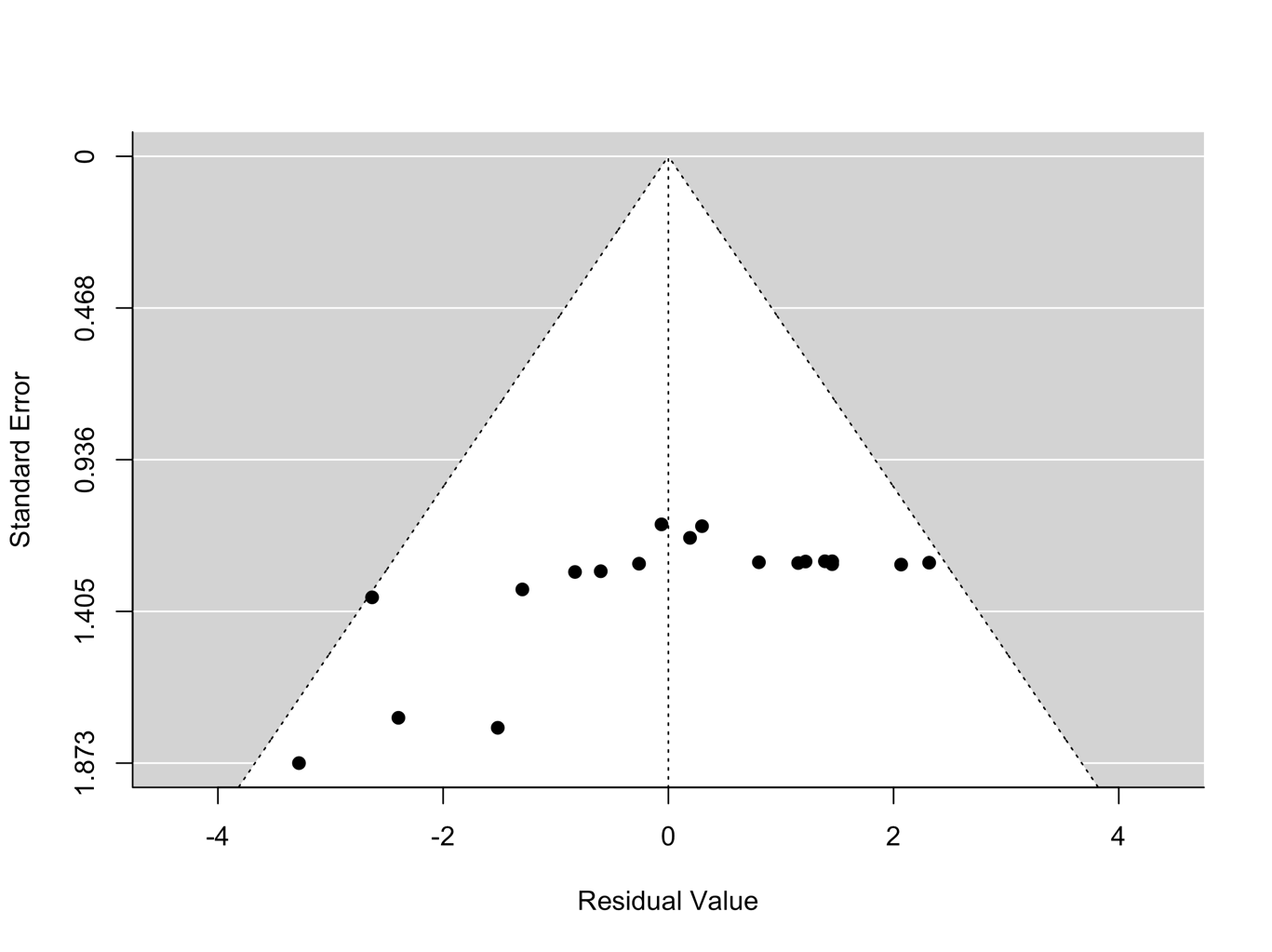


**Figure S3. Funnel plot for the meta-regression model for sexual violence from others’ drinking (main model, k = 19).**

## S18 – Intimate partner violence from the partner’s alcohol use: results from meta-regression models

**Table S15. Results from meta-regression models: intimate partner violence from the partner’s alcohol use (outcome).**

|  | **Estimate**  **(95% CI)** | **95% PI** | ***p*-value^‡^** | **Q (p-value)** | **I^2^** |
| --- | --- | --- | --- | --- | --- |
| **Main model without intercept (without intercept, k = 21)** | | | | | |
| Violence category: emotional | 2·7 (1·1-6·3) | 0·2-30·0 | ref | 2094·58 (< ·001) | 84·2 |
| Violence category: physical | 0·6 (0·2-1·3) | 0·0-8·0 | <·001 | . | . |
| Violence category: sexual | 0·4 (0·1-1·6) | 0·0-7·3 | ·004 | ·. | . |
| **Sensitivity analysis: accounting for risk of bias^*^ (without intercept, k = 21)** | | | | | |
| Violence category: emotional | 2·1 (0·3-12·3) | 0·1-37·0 | ref | 1784·87 (< ·001) | 86·0 |
| Violence category: physical | 0·4 (0·1-2·8) | 0·0-10·7 | <·001 | . | . |
| Violence category: sexual | 0·3 (0·0-2·8) | 0·0-9·5 | ·003 | . | . |
| **Sensitivity analysis: main model excluding studies reporting lifetime prevalence (Basile et al. 2021; without intercept, k = 20)** | | | | | |
| Violence category: emotional | 2·2 (1·0-4·6) | 0·2-18·2 | ref | 2040·12 (< ·001) | 78·2 |
| Violence category: physical | 0·5 (0·2-1·0) | 0·0-4·5 | <·001 | . | . |
| Violence category: sexual | 0·2 (0·0-0·8) | 0·0-2·4 | <·001 | . | . |
| **Sensitivity analysis: main model excluding studies having interviewed perpetrators (De Oliveira et al. 2009; without intercept, k = 19)** | | | | | |
| Violence category: emotional | 2·3 (1·0-5·5) | 0·2-18·2 | ref | 2046·49 (< ·001) | 81·2 |
| Violence category: physical | 0·5 (0·2-1·1) | 0·0-4·5 | <·001 | . | . |
| Violence category: sexual | 0·4 (0·1-1·5) | 0·0-2·4 | ·007 | . | . |

Note: CI = confidence interval. PI = prediction interval (estimated for models without intercept only). Ref = reference. *I*^2^ indicates between-study heterogeneity. Three-level model to account for clustering of data within individual studies, using study ID as cluster.^10^ ^‡^ significance of the comparison of each category with reference. ^*^ no significant difference between studies with low/moderate versus critical risk of bias, *p* = ·730.


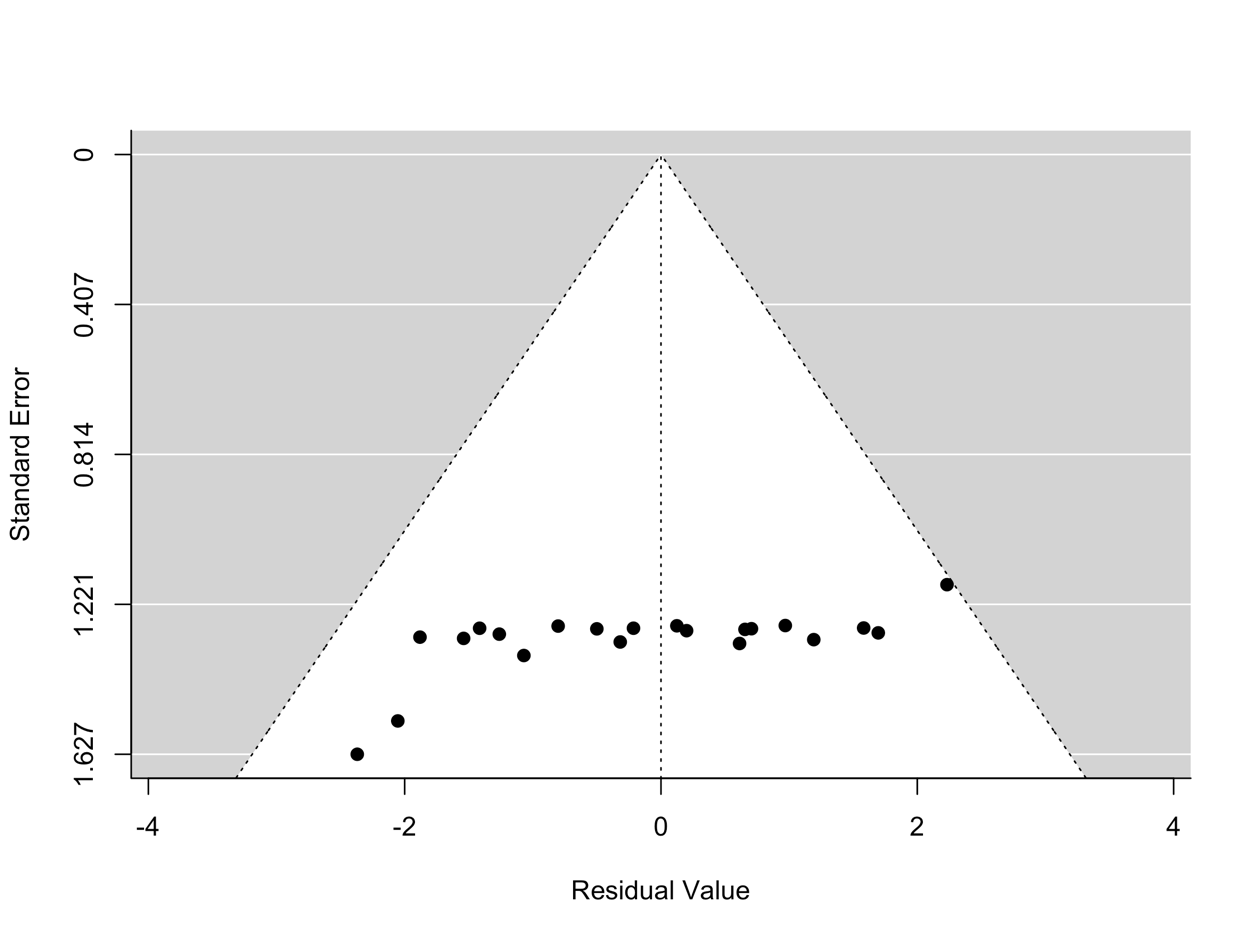


**Figure S4. Funnel plot for the meta-regression model for intimate partner violence from the partner’s alcohol use (main model, k = 21).**

## References

1 Institute for Health Metrics and Evaluation (IHME). Global Health Data Exchange: Countries. 2022. https://ghdx.healthdata.org/countries (accessed Sept 2, 2022).

2 Manthey J, Shield K, Rylett M, Hasan O, Probst C, Rehm J. Alcohol exposure between 1990 and 2017 and forecasts until 2030: A global modelling study. *The Lancet* 2019; **393**: 2493–502.

3 World Bank. GDP, PPP (current international $). International Comparison Program. Washington, DC: World Bank, 2022 https://data.worldbank.org/indicator/NY.GDP.MKTP.PP.CD (accessed Sept 2, 2022).

4 World Bank, Development Research Group. Gini index (World Bank estimate) [Data set]. 2022 https://data.worldbank.org/indicator/SI.POV.GINI?view=chart (accessed Sept 2, 2022).

5 OECD. Income inequality (indicator) [Data set]. 2022 https://data.oecd.org/inequality/income-inequality.htm (accessed Oct 28, 2022).

6 Census and Statistics Department. Thematic Report: Household Income Distribution in Hong Kong. 2016 Population By-Census (Report). 2017. https://www.censtatd.gov.hk/en/EIndexbySubject.html?pcode=D5321605&scode=459 (accessed Nov 4, 2022).

7 International Labour Organization. ILO Modelled Estimates and Projections database (ILOEST). ILOSTAT. Geneva, Switzerland: International Labor Organization, 2022 https://ilostat.ilo.org/data/ (accessed Sept 2, 2022).

8 United Nations, Department of Economic and Social Affairs, Population Division. World Population Prospects 2022, online edition. [Data set]. 2022. https://population.un.org/wpp/Download/Standard/Population/ (accessed Sept 2, 2022).

9 Ripley B, Venables B, Bates D, Hornik K, Gebhardt A, Firth D. Modern Applied Statistics with S. 2024. https://cran.r-project.org/web/packages/MASS/MASS.pdf (accessed Feb 22, 2024).

10 Harrer M, Cuijpers P, Furukawa T, Ebert D. Doing Meta-Analysis in R: A Hands-on Guide. Boca Raton, FL and London, UK: Chapman & Hall/CRC Press, 2021 https://bookdown.org/MathiasHarrer/Doing_Meta_Analysis_in_R/ (accessed Nov 15, 2022).
